# Evaluating the impact of COVID‐19 vaccination strategies on infections and hospitalisations in Victoria with non‐seasonal epidemic wave patterns: a modelling study

**DOI:** 10.5694/mja2.52677

**Published:** 2025-06-01

**Authors:** Fenella McAndrew, Romesh Abeysuriya, Nick Scott

**Affiliations:** ^1^ Burnet Institute Melbourne VIC; ^2^ Monash University Melbourne VIC

**Keywords:** Models, statistical, COVID‐19, Vaccination

## Abstract

**Objectives:**

To assess the impact of different coronavirus disease 2019 (COVID‐19) vaccination strategies on infections and hospitalisations in the context of non‐seasonal epidemic waves.

**Study design:**

Dynamic compartmental model‐based analysis.

**Setting:**

Victoria (Australia).

**Intervention:**

Alternative COVID‐19 vaccination strategies: baseline (low population coverage — 18–64 years, 7%; 65 years or older, 44% — vaccinations distributed evenly across the year); high coverage for all age groups, with vaccinations spread across year; increased coverage for people aged 65 years or older; annual vaccination campaigns that achieve coverage equivalent to that of influenza vaccinations (18–64 years, 25%; 65 years or older, 59%), commencing in March (same time as influenza vaccination campaign), August, or December; no further COVID‐19 vaccinations for people under 65 years of age; no further COVID‐19 vaccinations for anyone. Vaccination scenarios used different assumptions about COVID‐19 epidemic wave periodicity, and peak infections magnitude and month.

**Main outcome measures:**

Mean incidence of severe acute respiratory syndrome coronavirus 2 (SARS‐CoV‐2) infections and COVID‐19‐related hospitalisations over a ten‐year projection period.

**Results:**

The low baseline population level of recent COVID‐19 vaccination means that any increase in coverage could reduce infection and hospitalisation incidence. Increasing COVID‐19 vaccination coverage to match that of influenza vaccination with an annual vaccination campaign reduced the mean incidence of infections by 1–13% and that of hospitalisations by 3–14%, depending on the timing of vaccination campaigns with respect to the epidemic infections peak and assumptions about epidemic wave characteristics. Increasing coverage for people aged 65 years or older reduced hospitalisation incidence by 9–26%, but required twice as many vaccine doses as the annual campaign strategies.

**Conclusions:**

Annual COVID‐19 vaccination campaigns at the same time as those for influenza vaccination could reduce the number of COVID‐19‐related hospitalisations, with lower logistical requirements than alternative approaches.

**The known**: Despite COVID‐19 leading to 62 000 hospitalisations and 6500 deaths in Australia during 2023, only 11% of people aged 16 years or older had been vaccinated in the preceding twelve months. As COVID‐19 epidemics do not follow a regular seasonal pattern in Australia, it is unclear how annual vaccination programs should be organised.

**The new**: Our modelling study found that annual vaccination campaigns could reduce the annual number of SARS‐CoV‐2 infections by 1–13% and COVID‐19 hospitalisations by 3–14%.

**The implications**: Annual vaccination campaigns alongside those for influenza vaccination could reduce the number of COVID‐19 infections and the burden on hospitals.

Vaccines are a major component of the public health response to infectious diseases. The first coronavirus disease 2019 (COVID‐19) vaccines were developed within nine months of the World Health Organization declaration of the pandemic and were rapidly deployed to reduce the incidence of symptomatic and severe illness.^1^ Of the many vaccine types developed during the pandemic, mRNA vaccines were the most frequently used.^2^ The effectiveness of the mRNA vaccines wanes with time and differs by severe acute respiratory syndrome coronavirus 2 (SARS‐CoV‐2) variant,^3^, ^4^ but during the three months following vaccination they provide people who have previously been infected or received COVID‐19 vaccines about 75% protection against infection with the Omicron SARS‐CoV‐2 variant and 85% protection against severe disease.^5^, ^6^ This protection is important for people at particular risk of severe COVID‐19, including those over 65 years of age and people with other medical conditions.^7^, ^8^

COVID‐19 vaccination recommendations and eligibility criteria changed over time. In Australia, the initial two‐course COVID‐19 vaccination program commenced on 22 February 2021,^9^ with priority for people at greatest risk of severe disease (including residents of aged care facilities) or infection (people working in health care).^10^, ^11^ As the vaccine supply increased, eligibility was expanded to all people aged 16 years or older, and proof of vaccination was required for visiting many public venues.^12^ In October 2021, the Australian Technical Advisory Group on Immunisation (ATAGI) recommended a third vaccine dose for people with severe immunocompromise,^13^ and in November 2021 expanded the recommendation to all people aged 18 years or older.^14^ ATAGI has subsequently recommended boosters every six months for people aged 65 years or older, and every twelve months for people with risk factors for severe COVID‐19.^15^ Other people aged 18 years or older are eligible for COVID‐19 vaccination every twelve months, but ATAGI has not recommended that they should receive one.^15^

By April 2022, 95% of Australians aged 16 years or older had received at least two COVID‐19 vaccine doses.^16^ However, following reduced public promotion, the uptake of booster doses has been low;^17^, ^18^ in Victoria in December 2023, 50% of people aged 16 years or older had received third doses and 25% fourth doses.^19^ By July 2024, only 7% of Australians aged 18–64 years and 44% of people aged 65 years or older had received COVID‐19 vaccine doses during the past twelve months.^20^

Influenza vaccination campaigns are scheduled for winter each year to maximise their impact, given the seasonal nature of epidemic waves.^21^, ^22^ In Australia, government advertising, workplace promotions, and free vaccination for people at particular risk of severe influenza means that annual population coverage is typically about 30%.^23^ Unlike influenza, epidemic waves of COVID‐19 have not been seasonal, and in Australia during 2021–2024 have occurred at about six‐month intervals corresponding to the emergence of new SARS‐CoV‐2 variants.^24^

The optimal allocation of vaccine doses by population group was investigated when COVID‐19 vaccines were first introduced.^25^, ^26^, ^27^ In contrast, COVID‐19 vaccination strategies in the context of low vaccine uptake, non‐seasonal epidemic waves, and waning immunity from both vaccination and past infection have not been assessed. We therefore modelled the impact of different COVID‐19 vaccination strategies, with the aim of providing insights for future vaccination guidelines.

## Methods

The setting for our dynamic compartmental model study was Victoria, a state with a population of 6.6 million people in 2022, 17% of whom were over 65 years of age and 60% aged 18–65 years.^28^ Age‐specific risks of COVID‐19, severe COVID‐19, hospitalisation, and death by immunity status (vaccination or exposure‐acquired, including waning of immunity) were derived from published reports, as described previously.^19^, ^29^ Vaccination and COVID‐19 treatment coverage data were obtained from the Victorian Department of Health (Supporting Information, section 1.1). The model was calibrated to 2022 Victorian hospital and wastewater data on the relative circulating prevalence of SARS‐CoV‐2 variants.^30^

### Model overview

We used Atomica, a Python package for building compartmental models (https://github.com/atomicateam/atomica). COVID‐19 vaccination scenarios and outcomes in Victoria were projected using a dynamic compartmental SEIR (susceptible, exposed, infectious, recovered) model (Box 1). The model population was stratified by age group (0–5, 6–17, 18–64, 65–79, 80 years or older) to assess differences in disease outcomes, vaccination uptake, and vaccination guidelines. Model compartments were also stratified to include multiple SARS‐CoV‐2 variants and took cross‐immunity into account. The model included births, all‐cause and COVID‐19‐specific deaths, and ageing as parameters, but a constant population size was assumed because our projections were intended to cover the near future (further details: Supporting Information, section 1.2).^31^

Box 1 Framework of our dynamic compartmental model of the impact of vaccination strategies on the incidence of SARS‐CoV‐2 infections and COVID‐19‐related hospitalisations in Victoria*

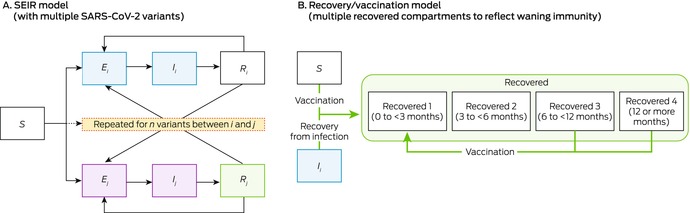

COVID‐19 = coronavirus disease 2019; SARS‐CoV‐2 = severe acute respiratory syndrome coronavirus 2; SEIR = susceptible (S), exposed (E), infectious (I), recovered (R); subscript i = variant i.

* Further details: Supporting Information, table 1.

### Immunity

Given the high level of COVID‐19 vaccination coverage among Australians aged 18 years or older by 2022, and multiple COVID‐19 waves in Victoria, it is likely that most people have hybrid immunity (acquired from vaccine and virus exposure); information about the population distribution of immunity is inadequate for stratifying the model by immunity type. We therefore considered infection or vaccination as equivalent immune‐boosting events, the effects of which wane over time (further details: Supporting Information, section 1.2); in a sensitivity analysis, we assumed that vaccination provided less protection than infection. To reflect waning immunity following immune‐boosting events, the model includes four recovered compartments corresponding to the time since the most recent immune‐boosting event (0 to less than three, three to less than six, six to less than twelve, twelve or more months); the level of protection against subsequent infections declines with time (Box 1).

### SARS‐CoV‐2 variants

When a new SARS‐CoV‐2 variant is introduced into the model, recovered compartments corresponding to people infected with or vaccinated against earlier variants are modelled to have lower protection against infection with the new variant than against earlier variants. The difference in protection is based on a set of cross‐immunity parameters. All modelled new variants were assumed to be Omicron subvariants, with the same incubation period, infectious period, and severity of outcomes (further details: Supporting Information, section 1.2).

The timing and size of future SARS‐CoV‐2 variant epidemic waves is unknown. For our baseline scenario, we assumed epidemic waves at six‐month intervals of similar magnitude to that of the XBB epidemic wave in Victoria during November 2022 – February 2023; we also tested different assumptions about the periodicity and magnitude (peak number of new infections) of the epidemic waves. The six‐month period was selected to approximate the epidemiology of COVID‐19 during 2022–2024 (six Omicron subvariants caused five distinct waves during December 2021 – January 2024^24^); the magnitude of the XBB epidemic wave was selected as a balance between those of early Omicron epidemic waves, during which population immunity was low, and later epidemic waves, when case ascertainment was lower because reporting requirements for hospitalised cases were relaxed.^32^

### Calibration and initialisation

The model was initialised with an entirely susceptible population and a constant baseline age‐specific vaccination rate equivalent to that of Victoria in July 2024 (Supporting Information, section 1.2). The model was run until the outputs reached a steady epidemic wave pattern, with epidemic waves every six months and new infection peaks similar to that of the 2022–23 XBB wave in Victoria. All scenarios were run for fifteen years; the simulation reached a steady pattern during the first five years, and we projected outcomes for the following ten‐year period, chosen to ensure that the projections were not influenced by the chosen start or end point of the projection period.

An optimisation algorithm was used to calibrate the model; parameters were varied for the force of infection proportionality constant, cross‐immunity between SARS‐CoV‐2 variants, cases seeded when introducing a new variant, and mean duration of infection (as a proxy for the impact of isolation), such that the model outputs were aligned with the following outcomes during the 2022–23 XBB epidemic wave in Victoria: cumulative number of hospitalisations and peak daily hospital admissions number (Supporting Information, section 1.5), SARS‐CoV‐2 infection incidence per 100 000 person‐years (estimated in earlier model‐fitting exercises^19^), and the rate of variant displacement (time in which proportion of infections with the new variant grew from 5% to 20%, based on Victorian wastewater data) (further details: Supporting Information, part 2).

### Scenarios

The modelled scenarios are summarised in Box 2. The baseline scenario (low coverage, vaccinations uniformly spread across year) reflects current levels of COVID‐19 vaccination coverage in Victoria. The three scenarios including an annual vaccination campaign — commencing in different months to assess the interaction of campaign timing with assumptions about epidemic wave patterns — were modelled as having similar rollout speed and achieving similar age‐specific and time‐varying coverage as the Victorian 2024 influenza vaccination campaign (Supporting Information, section 1.6).^23^ In the modelled scenarios, people under 18 years of age did not receive additional vaccines because they are not eligible for COVID‐19 vaccine boosters in Australia. As future epidemic wave patterns are unknown, all vaccination scenarios were run with several sets of different assumptions regarding the periodicity of epidemic waves — six months (baseline), five months, eight months, or twelve months (with infection peaks in March, July, or November) — and epidemic wave peak infection level — XBB‐like (baseline), 30% lower peak than XBB, 15% higher peak than XBB. The periodicity and magnitude of the epidemic waves in these scenarios are not intended to be forecasts.

Box 2 **Proportion of population who received booster COVID‐19 vaccine doses annually by age group and scenario**

**Table:** 

Scenario	Age group (years)				
	0–5	6–17	18–64	65–79	80 or older
Low coverage, vaccinations spread across year (baseline)*	0	0	6%	38%	22%
(1) High coverage, vaccinations spread across year	0	0	57%	133%^†^	76%
(2) Increased coverage for people aged 65 years or older	0	0	6%	133%^†^	76%
(3‐5) Annual campaign scenarios (commencing March, July, or November)^‡^	0	0	25%	59%	59%
(6) No vaccination of people under 65 years of age	0	0	0	38%	22%
(7) No vaccination (any age group)	0	0	0	0	0
(8) Doubling coverage for those under 65 years old	0	0	12%	38%	22%
(9) Coverage equivalent to influenza vaccination coverage, uniform through year	0	0	25%	59%	59%

COVID‐19 = coronavirus disease 2019.

* Based on COVID‐19 vaccination coverage in Victoria, 2024.^32^

† Some people receive more than one vaccine dose within twelve‐month period, in accordance with recommendation for boosters every six months.

‡ Assumed to achieve same age group‐based coverage as for influenza vaccination.

### Outcomes and uncertainty

After the model reached a stable epidemic wave pattern, mean numbers of infections and hospitalisations per 100 000 person‐years (with 95% confidence intervals, CIs) were projected over the ten‐year period for each scenario.

A multivariate probabilistic uncertainty analysis used uncertainty intervals for population size, births, age‐specific all‐cause mortality rates, COVID‐19 mortality rates, and vaccine efficacy parameters (Supporting Information, sections 1.1 and 1.4). For each scenario and epidemic wave characteristic assumption set, the model was run fifty times and bootstrapping methods were used to generate mean values with 95% CIs for the mean differences in incidence of infections and hospitalisations, and in the annual vaccine doses delivered.

### Ethics approval

We did not seek ethics approval for our modelling study based on publicly available data.

## Results

The model was highly sensitive to the start and end of the measurement period because of variations in epidemic wave periodicity and magnitude. The results converged and were less sensitive to the choice of start or end points if the model was run for ten years rather than one or five years; each scenario was therefore projected over a 10‐year period (Supporting Information, part 3).

After calibration of the baseline scenario — no changes to current vaccination coverage, new SARS‐CoV‐2 variants emerging every six months, epidemic waves of magnitude equivalent to the XBB epidemic wave — we projected a mean incidence of 118 288 SARS‐CoV‐2 infections (95% CI, 104 221–143 107) (Supporting Information, table 4) and 326 COVID‐19‐related hospitalisations (95% CI, 228–383) per 100 000 person‐years (Supporting Information, table 5) during the ten‐year projection period, and a total of 10 755 hospitalisations per epidemic wave. For each epidemic wave, the projected number of hospitalisations was consistent with the number during the XBB wave in Victoria (11 098 hospitalisations), as was the time taken for new SARS‐CoV‐2 variants to displace earlier variants (Box 3).

Box 3 **COVID‐19‐related hospitalisations during COVID‐19 waves in Victoria, historical (A) and projected by our model (B, C); time required for replacement by new SARS‐CoV‐2 variants, historical and projected by our model (D)**

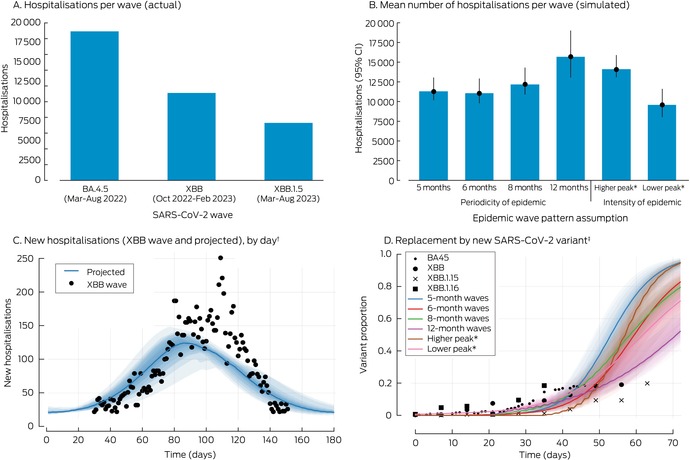

CI = confidence interval; COVID‐19 = coronavirus disease 2019; SARS‐CoV‐2 = severe acute respiratory syndrome coronavirus 2.

* Total number of infections for epidemic wave, relative to XBB wave, Victoria, 2022–23: lower = 30% fewer cases; higher = 15% more cases.

† Assumptions for projected hospitalisations: infection waves every six months, infection peak equivalent to XBB wave, Victoria, 2022–23. The shaded regions correspond to the uncertainty analysis quantiles (55th to 95th).

‡ Time taken for new variant proportion to grow from 5% to 20%, calibrated using Victorian wastewater data for historical SARS‐CoV‐2 variant waves. The shaded regions correspond to the uncertainty analysis quantiles (55th to 95th).

With different epidemic wave pattern assumptions, the mean incidence of infections ranged from about 78 000 (twelve‐month epidemic waves; Supporting Information, table 4) to 150 243 per 100 000 person‐years (five‐month epidemic waves; Supporting Information, table 4); the mean incidence of hospitalisations ranged from about 223 (twelve‐month epidemic waves; Supporting Information, table 5) to 403 per 100 000 person‐years (five‐month epidemic waves; Supporting Information, table 5).

### Vaccination scenarios

In scenarios in which people under 65 years of age were not vaccinated (scenario 6) or the vaccination coverage among people under 65 years of age was twice that in the baseline scenario (scenario 8) the mean incidence of infections (Box 4) and hospitalisations (Box 5) did not change, irrespective of epidemic wave assumptions, because the current level of vaccination coverage for people under 65 years of age is low.

Box 4 Proportional changes in projected mean annual incidence of SARS‐CoV‐2 infections per 100 000 person‐years and annual number of COVID‐19 vaccine doses administered, compared with baseline, by vaccination scenario and epidemic wave characteristics*

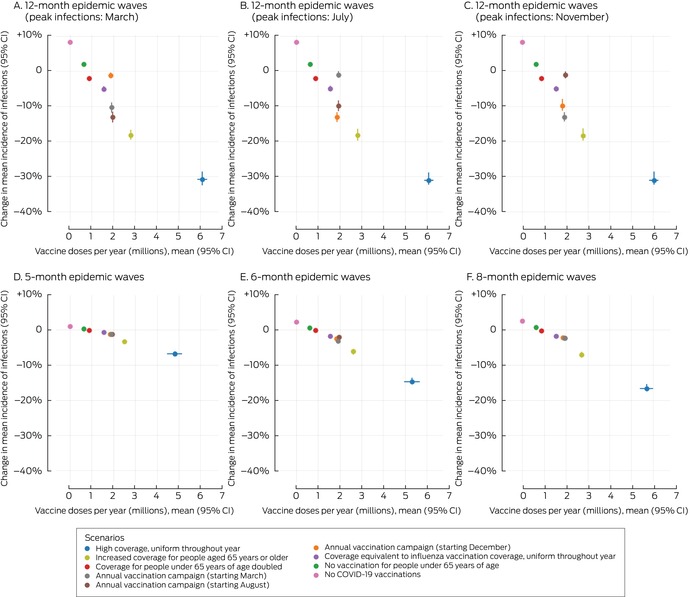

* The infections data for these graphs are included in the Supporting Information, table 4.

CI = confidence interval; COVID‐19 = coronavirus disease 2019; SARS‐CoV‐2 = severe acute respiratory syndrome coronavirus 2.

Box 5 Proportional changes in projected mean annual incidence of total COVID‐19‐related hospitalisations per 100 000 person‐years and annual number of COVID‐19 vaccine doses administered, compared with baseline, by vaccination scenario and epidemic wave characteristics*

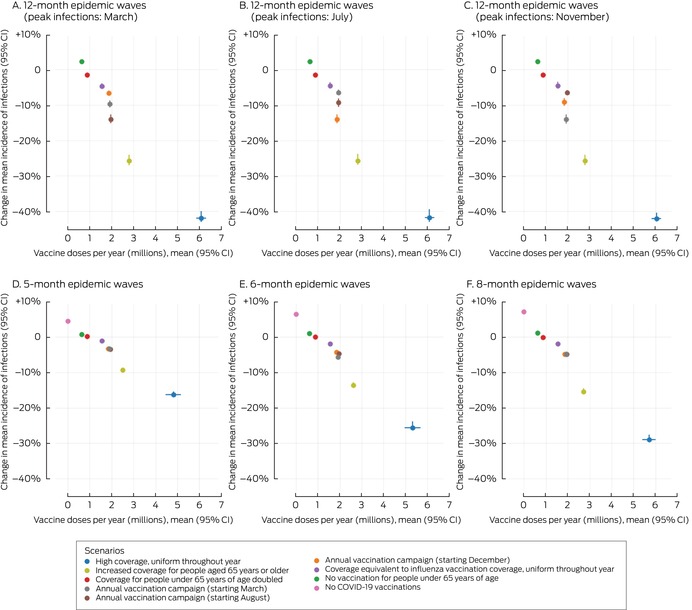

* The hospitalisation data for these graphs are included in the Supporting Information, table 5.

CI = confidence interval; COVID‐19 = coronavirus disease 2019.

In the scenario in which no‐one was vaccinated (scenario 7) the mean incidence of infections increased by 1–8% (Box 4) and that of hospitalisations by 5–13% (Box 5), depending on epidemic wave assumptions, primarily because the current level of vaccination coverage for people aged 65 years or more is high (Box 2), and the probability of hospitalisation following infection is greater for this age group than for younger people (Supporting Information, table 2).

In scenarios with annual vaccination campaigns (scenarios 3 to 5), the mean incidence of infections (1–13% reduction) and hospitalisations (3–14% reduction) were each lower than in the baseline scenario, regardless of campaign timing or epidemic wave assumptions. With twelve‐month epidemic waves, the mean incidence of infections was 1–13% lower and that of hospitalisations 6–14% lower than in the baseline scenario, depending on the peak infection month; campaigns commencing about six months before the infection peak were most effective. With five‐, six‐, or eight‐month epidemic waves, similar reductions in the mean incidence of infections (1–3%) and hospitalisations (3–6%) were projected, irrespective of vaccination campaign timing (Box 6). Increasing COVID‐19 vaccination coverage to match that of influenza vaccination coverage, but with COVID‐19 vaccinations distributed uniformly throughout the year, (scenario 9) achieved reductions in infections (1–5%) and hospitalisations (1–5%) that were smaller than in the annual COVID‐19 vaccination campaign scenarios (scenarios 3 to 5) (Box 4, Box 5).

Box 6 Proportional changes in projected mean annual incidence of SARS‐CoV‐2 infections and total COVID‐19‐related hospitalisations per 100 000 person‐years, compared with baseline, by vaccination campaign scenario and epidemic wave characteristics*

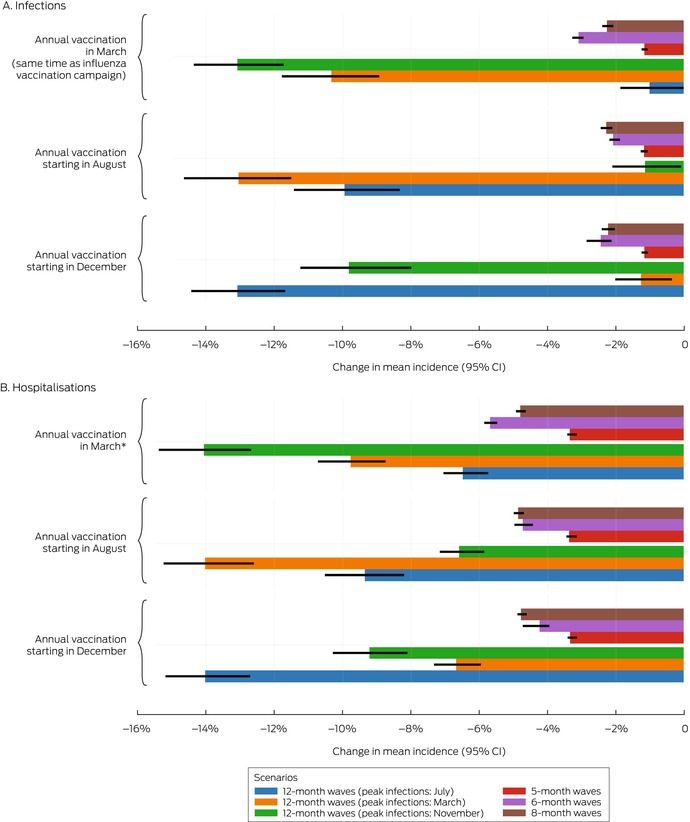

* The data for these graphs are included in the Supporting Information, tables 4 and 5.

CI = confidence interval; COVID‐19 = coronavirus disease 2019; SARS‐CoV‐2 = severe acute respiratory syndrome coronavirus 2.

High uniform coverage throughout the year (scenario 1) resulted in the greatest reduction in mean incidence of infections (7–31%) and hospitalisations (16–42%) with all epidemic wave assumptions, as well as the greatest number of vaccine doses delivered (about six million per year) (Box 4, Box 5). Increasing vaccine coverage among people aged 65 years or older reduced the mean incidence of infections (3–18%) and hospitalisations (9–26%) (scenario 2), but required twice as many vaccine doses as the annual campaign strategies (scenario 3 to 5) (Box 4, Box 5).

### Sensitivity analyses

If vaccination was assumed to provide less protection than infection, the impact of vaccination was reduced in all scenarios (Supporting Information, section 4.1). The ranking of the effectiveness of the vaccination scenarios was not influenced by assumptions about epidemic wave magnitude, and the effectiveness of all annual vaccination campaigns (scenarios 3 to 5) was similar (Supporting Information, section 4.2). The proportional reductions in the numbers of infections and hospitalisations in each scenario were larger for lower magnitude epidemic waves and smaller for greater magnitude epidemic waves (Supporting Information, section 4.2).

## Discussion

We used a compartmental model to simulate COVID‐19 waves in Victoria to assess the effects of various COVID‐19 vaccination strategies, including increasing vaccination coverage throughout the year and timed vaccination campaigns. The study setting was Victoria because historical COVID‐19 data were readily available, but our findings could be applicable to other Australian states and other high income countries with vaccination coverage and population age structure similar to those of Victoria. Further analysis would be required for countries in which the availability of COVID‐19 vaccines is more restricted or demographic characteristics are markedly different.

In the counterfactual scenario of no vaccination of people under 65 years of age, the incidence of infections and hospitalisations was only slightly higher than in the baseline scenario, which suggests that the current low COVID‐19 vaccination coverage in this age group (7% vaccinated in the past twelve months^20^) has little impact at the population level on infection and hospitalisation rates. Vaccination against COVID‐19 is not currently required for anyone in Australia,^15^ and vaccine boosters are recommended for people aged 18–64 years only if they have specific medical conditions or work with people at high risk of serious COVID‐19 illness.^33^ This contrasts with influenza; despite a considerably smaller disease burden (376 deaths and 3696 hospitalisations during the 2023 influenza season;^34^ COVID‐19: 6500 deaths and 62 000 hospitalisations during 2023 [Supporting Information, section 1.5]^32^) and a less effective vaccine (influenza: 44% protection against hospitalisation;^35^ COVID‐19: 85% protection against severe disease^6^), annual vaccine campaigns achieve about 32% coverage among people aged six months or older.^23^ This difference, and the low number of COVID‐19 vaccine doses distributed, indicate that a specific vaccination campaign could reduce the COVID‐19 burden.

Our modelling indicates that increasing vaccination coverage among people over 65 years of age could reduce the incidence of SARS‐CoV‐2 infections and COVID‐19‐related hospitalisations. COVID‐19 vaccine acceptance among people over 65 years of age is high,^36^, ^37^ and in July 2024 about 44% of Australians in this age group had received COVID‐19 vaccine doses during the preceding twelve months.^20^ Nevertheless, our model indicates that further increasing coverage would be beneficial, although increasing coverage in this age group could be difficult. Moreover, as people aged 65 years or older who have not already received vaccine boosters comprise only a small proportion of the total population, limiting a vaccine strategy to this age group might not achieve the greatest population‐level effects possible.

Our model suggests that starting COVID‐19 vaccination campaigns in March, at the same time as influenza vaccination campaigns, could be effective in reducing numbers of SARS‐CoV‐2 infections and COVID‐19‐related hospitalisations, with the degree of benefit depending on assumptions about epidemic wave patterns. In scenarios in which COVID‐19 vaccination coverage among people aged 18 years or older was equivalent to that for influenza vaccination, the mean incidence of infections was reduced by 1–13% and that of hospitalisations by 3–14%, compared with the baseline scenario. If this finding can be extrapolated to all of Australia, it would be equivalent to averting the number of hospitalisations attributed to an entire influenza season. In scenarios with COVID‐19 epidemic waves at twelve‐month intervals, the timing of vaccination campaigns had only a small effect on hospitalisations. For scenarios including multiple COVID‐19 waves each year, reflecting the epidemiology of COVID‐19 during 2021–2023, reductions in hospitalisation incidence in scenarios with annual vaccination campaigns were similar, irrespective of the magnitude or timing of the infection waves.

### Limitations

Firstly, in the absence of data on the distribution of immunity acquired during previous infections and vaccinations, vaccines and infections were modelled as providing equivalent protection from SARS‐CoV‐2 infection. Secondly, we split the model population into age groups, but did not consider subgroups of people with other medical conditions or who were more hesitant about vaccination.^38^ Further exploration of the benefits of vaccination campaigns for specific groups of people at greater risk of infection or severe disease than the general population could aid vaccination strategy development. Thirdly, our compartmental model did not reflect the heterogeneity of the Victorian population (apart from by age group); moreover, we assumed homogeneous mixing between age groups. This means that the model captures the impact on herd immunity, but may underestimate it for older age groups if they exhibited more assortative mixing. Fourthly, we recorded the number of vaccine doses distributed, but did not consider the costs of different scenarios.

### Conclusion

Because population COVID‐19 vaccination coverage is currently low, any increase in coverage could reduce the numbers of infections and hospitalisations. Introducing annual COVID‐19 vaccination campaigns alongside those for influenza vaccination could reduce the incidence of COVID‐19 hospitalisations by 3–14% without the logistical challenges of alternative approaches.

## Open access

Open access publishing facilitated by Monash University, as part of the Wiley ‐ Monash University agreement via the Council of Australian University Librarians.

## Competing interests

No relevant disclosures.

## Data sharing

Data sharing is not applicable to this article as no new data were generated.

## Code sharing

The code used for our analysis is available in Zenodo: https://doi.org/10.5281/zenodo.14915339 (24 Feb 2025).

## Supporting information


Supplementary methods and results

## References

[1] European Medicines Agency . EMA recommends first COVID‐19 vaccine for authorisation in the EU [media release]. 21 Dec 2020. https://www.ema.europa.eu/en/news/ema‐recommends‐first‐covid‐19‐vaccine‐authorisation‐eu (viewed Apr 2025).

[2] Mathieu E , Ritchie H , Rodés‐Guirao L , et al. Coronavirus (COVID‐19) vaccinations. Our World in Data; undated. https://ourworldindata.org/covid‐vaccinations (viewed Apr 2025).

[3] Menegale F , Manica M , Zardini A , et al. Evaluation of waning of SARS‐CoV‐2 vaccine‐induced immunity: a systematic review and meta‐analysis. JAMA Netw Open 2023; 6: e2310650. 37133863 10.1001/jamanetworkopen.2023.10650 PMC10157431

[4] Khoury DS , Docken SS , Subbarao K , et al. Predicting the efficacy of variant‐modified COVID‐19 vaccine boosters. Nat Med 2023; 29: 574‐578. 36864253 10.1038/s41591-023-02228-4

[5] Bobrovitz N , Ware H , Ma X , et al. Protective effectiveness of previous SARS‐CoV‐2 infection and hybrid immunity against the omicron variant and severe disease: a systematic review and meta‐regression. Lancet Infect Dis 2023; 23: 556‐567. 36681084 10.1016/S1473-3099(22)00801-5 PMC10014083

[6] Arabi M , Al‐Najjar Y , Sharma O , et al. Role of previous infection with SARS‐CoV‐2 in protecting against omicron reinfections and severe complications of COVID‐19 compared to pre‐omicron variants: a systematic review. BMC Infect Dis 2023; 23: 432. 37365490 10.1186/s12879-023-08328-3 PMC10294418

[7] Sadarangani M , Abu Raya B , Conway JM , et al. Importance of COVID‐19 vaccine efficacy in older age groups. Vaccine 2021; 39: 2020‐2023. 33736921 10.1016/j.vaccine.2021.03.020 PMC7938751

[8] Choi WS , Cheong HJ . COVID‐19 vaccination for people with comorbidities. Infect Chemother 2021; 53: 155‐158. 34409789 10.3947/ic.2021.0302 PMC8032917

[9] Australian National Audit Office . Australia's COVID‐19 vaccine rollout. 17 Aug 2022. https://www.anao.gov.au/work/performance‐audit/australia‐covid‐19‐vaccine‐rollout (viewed Apr 2025).

[10] Prime Minister of Australia . National Cabinet statement [media release]. 28 June 2021. https://pmtranscripts.pmc.gov.au/release/transcript‐44077 (viewed Apr 2025).

[11] Chief Health Officer (Victoria) . Advice relating to the making of pandemic orders as required by section 165AL of the *Public Health and Wellbeing Act* 2008. 29 Aug 2022. https://www.health.vic.gov.au/publications/chief‐health‐officer‐advice‐to‐premier (viewed Apr 2025).

[12] Lowrey T. The COVID‐19 “vaccine passport” is coming. Here's how it could work and how it's legal. ABC News, 5 Aug 2021. https://www.abc.net.au/news/2021‐08‐05/covid‐vaccination‐passport‐how‐it‐could‐work/100350778 (viewed Apr 2025).

[13] Australian Technical Advisory Group on Immunisation . Recommendations on the use of a 3rd primary dose of COVID‐19 vaccine in individuals who are severely immunocompromised. Updated 1 Mar 2023. https://www.eviq.org.au/getmedia/4c0f9f3f‐a290‐49a9‐a22a‐198fc4f54d47/atagi‐recommendations‐on‐the‐use‐of‐a‐third‐primary‐dose‐of‐covid‐19‐vaccine‐in‐individuals‐who‐are‐severely‐immunocompromised.pdf (viewed Apr 2025).

[14] Minister for Health (Australia]. Start of COVID‐19 booster vaccination program [media release]. 8 Nov 2021. https://www.health.gov.au/ministers/the‐hon‐greg‐hunt‐mp/media/start‐of‐covid‐19‐booster‐vaccination‐program (viewed Apr 2025).

[15] Australian Department of Health and Aged Care . COVID‐19 vaccine advice and recommendations. Updated 20 Nov 2024. https://www.health.gov.au/our‐work/covid‐19‐vaccines/getting‐your‐vaccination/booster‐doses (viewed Apr 2025).

[16] Australian Department of Health and Aged Care . COVID‐19 vaccine rollout updates: 1 April 2022. https://www.health.gov.au/resources/publications/covid‐19‐vaccine‐rollout‐update‐1‐april‐2022?language=en2022 (viewed Apr 2025).

[17] Kleitman S , Fullerton DJ , Law MKH , et al. The psychology of COVID‐19 booster hesitancy, acceptance and resistance in Australia. Vaccines (Basel) 2023; 11: 907. 37243011 10.3390/vaccines11050907 PMC10222735

[18] Premier of Victoria . Giving employers options to keep workers COVIDsafe [media release]. 6 July 2022. https://www.premier.vic.gov.au/giving‐employers‐options‐keep‐workers‐covidsafe (viewed Apr 2025).

[19] McAndrew F , Abeysuriya RG , Sacks‐Davis R , et al. Admission screening testing of patients and staff N95 respirators are cost‐effective in reducing COVID‐19 hospital‐acquired infections. J Hosp Infect 2024; 152: 81‐92. 39019117 10.1016/j.jhin.2024.06.015

[20] Australian Department of Health and Aged Care . COVID‐19 vaccine rollout updates: 12 July 2024. https://www.health.gov.au/resources/publications/covid‐19‐vaccine‐rollout‐update‐12‐july‐2024 (viewed Apr 2025).

[21] Australian Department of Health and Aged Care . ATAGI statement on the administration of seasonal influenza vaccines in 2024. Updated 16 July 2024. https://www.health.gov.au/resources/publications/atagi‐statement‐on‐the‐administration‐of‐seasonal‐influenza‐vaccines‐in‐2024 (viewed Apr 2025).

[22] Australian Department of Health and Aged Care . 2024 National Immunisation Program influenza vaccination: early advice for health professionals. 29 Feb 2024. https://www.health.gov.au/news/2024‐national‐immunisation‐program‐influenza‐vaccination‐early‐advice‐for‐health‐professionals (viewed Apr 2025).

[23] National Centre for Immunisation Research and Surveillance . Historical influenza vaccine coverage (%) at end of year, by age group, Australia, 2020–2024. Updated Apr 2025. https://ncirs.org.au/influenza‐vaccination‐coverage‐data/all‐persons‐2020‐2025‐ytd‐influenza‐vaccination‐coverage (viewed Apr 2025).

[24] Australian Department of Health and Aged Care . COVID‐19 Australia: epidemiology report 84 reporting period ending 11 February 2024. 23 Apr 2024. https://www1.health.gov.au/internet/main/publishing.nsf/Content/covid‐19_epidemiology_reports_australia_2022‐23.htm (viewed Apr 2025).

[25] McBryde ES , Meehan MT , Caldwell JM , et al. Modelling direct and herd protection effects of vaccination against the SARS‐CoV‐2 Delta variant in Australia. Med J Aust 2021; 215: 427‐432. https://www.mja.com.au/journal/2021/215/9/modelling‐direct‐and‐herd‐protection‐effects‐vaccination‐against‐sars‐cov‐2 34477236 10.5694/mja2.51263 PMC8662033

[26] MacIntyre CR , Costantino V , Trent M . Modelling of COVID‐19 vaccination strategies and herd immunity, in scenarios of limited and full vaccine supply in NSW, Australia. Vaccine 2022; 40: 2506‐2513. 33958223 10.1016/j.vaccine.2021.04.042 PMC8064825

[27] Fadaki M , Abareshi A , Far SM , Lee PT . Multi‐period vaccine allocation model in a pandemic: a case study of COVID‐19 in Australia. Transp Res E Logist Transp Rev 2022; 161: e102689. 10.1016/j.tre.2022.102689 PMC8995313 35431604

[28] Australian Bureau of Statistics . Regional population by age and sex, 2023. 29 Aug 2024. https://www.abs.gov.au/statistics/people/population/regional‐population‐age‐and‐sex/latest‐release (viewed Apr 2025).

[29] Scott N , Abeysuriya RG , Delport D , et al. COVID‐19 epidemic modelling for policy decision support in Victoria, Australia 2020–2021. BMC Public Health 2023; 23: 988. 37237343 10.1186/s12889-023-15936-w PMC10219801

[30] Coronavirus Victoria . Victorian wastewater testing results: October 2022. DataVic; updated 3 Jan 2025. https://discover.data.vic.gov.au/dataset/victorian‐wastewater‐testing‐results (viewed Apr 2025).

[31] McAndrew F , Abeysuriya RG , Scott N . Modelling the potential impact of annual COVID‐19 vaccination campaigns in the context of non‐seasonal epidemic waves; version 1. Zenodo, 24 Feb 2025. 10.5281/zenodo.14915339 (viewed Apr 2025).

[32] Australian Department of Health and Aged Care . Monitoring and reporting on COVID‐19. Updated 17 Oct 2024. Archived: https://web.archive.org/web/20241105022219/https://www.health.gov.au/topics/covid‐19/reporting (viewed Nov 2024).

[33] Department of Health and Aged Care . Statement on the administration of COVID‐19 vaccines in 2024. 29 Feb 2024. https://www.health.gov.au/sites/default/files/2024‐03/atagi‐statement‐on‐the‐administration‐of‐covid‐19‐vaccines‐in‐2024.pdf (viewed Apr 2025).

[34] Australian Department of Health and Aged Care . Australian influenza surveillance report: 2023 end of season summary. Dec 2023. https://www.health.gov.au/sites/default/files/2023‐12/aisr‐2023‐national‐influenza‐season‐summary.pdf (viewed Apr 2025).

[35] Guo J , Chen X , Guo Y , et al. Real‐world effectiveness of seasonal influenza vaccination and age as effect modifier: a systematic review, meta‐analysis and meta‐regression of test‐negative design studies. Vaccine 2024; 42: 1883‐1891. 38423813 10.1016/j.vaccine.2024.02.059

[36] Christou‐Ergos M , Bleicher K , Leask J . Factors associated with vaccination intention and uptake over time in a sample of older Australians. Vaccine 2024; 42: 3601‐3606. 38704261 10.1016/j.vaccine.2024.04.069

[37] Edwards B , Biddle N , Gray M , Sollis K . COVID‐19 vaccine hesitancy and resistance: correlates in a nationally representative longitudinal survey of the Australian population. PLoS One 2021; 16: e0248892. 33760836 10.1371/journal.pone.0248892 PMC7990228

[38] Aitken Z , Emerson E , Kavanagh AM . COVID‐19 vaccination coverage and vaccine hesitancy among Australians with disability and long‐term health conditions. Health Promot J Austr 2023; 34: 895‐902. 36565293 10.1002/hpja.691 PMC9880664

