# Supplementary material for: Evaluating the impact of COVID‐19 vaccination strategies on infections and hospitalisations in Victoria with non‐seasonal epidemic wave patterns: a modelling study
Source: Med J Aust. 2025 Jun 1;222(11):558–66. doi: 10.5694/mja2.52677 (PMC12167612; doi:10.5694/mja2.52677)
Supplement: Supplementary file 1 — Supplementary methods and results [file MJA2-222-558-s001.pdf]

MJA
The Medical Journal of Australia

Supporting Information

Supplementary methods and results

**This appendix was part of the submitted manuscript and has been peer reviewed.
It is posted as supplied by the authors.**

Appendix to: McAndrew F, Abeysuriya R, Scott N. Evaluating the impact of COVID-19 vaccination strategies on infections and hospitalisations in Victoria with non-seasonal epidemic wave patterns: a modelling study. *Med J Aust* 2025; doi: 10.5694/mja2.52677.

Supplementary methods

1. Model inputs

1.1. Model compartments and parameters

Table 1. Model compartments and stratification

Label	Model stratification	Description
a	Age	Superscript a representing compartments that have been stratified by age, for age category a ∈ A={0-5 years, 6-17 years, 18-64 years, 65-79 years, 80+ years}
v	Variant	Superscript v representing compartments that have been stratified by variant for $v \in V = \{v_1, v_2, \dots, v_n\}$
Label	Compartment	Description
$S^{a,v}$	Susceptible	People who have never been infected or vaccinated
$E^{a,v}$	Exposed	People who have recently been infected, but are not infectious, from the susceptible or recovered compartments
$I^{a,v}$	Infected	People who are infectious
$R_1^{a,v}$	Recovered 0-3 months	People 0-3 months since their last immune boosting event
$R_2^{a,v}$	Recovered 3-6 months	People 3-6 months since their last immune boosting event
$R_3^{a,v}$	Recovered 6-12 months	People 6-12 months since their last immune boosting event
$R_4^{a,v}$	Recovered 12 months	People 12+ months since their last immune boosting event

Table 2. Model parameters, including symbols used in the equations in the Supporting Information file, parameter name, value and source

Symbol	Parameter	Value	Source
κ	Cross immunity	0.1 (baseline)	Determined by calibration (Supporting Information, part 2)
$\lambda^v(t)$	Force of infection for variant v	A function described in section 1.2, *Model equations,* which is the prevalence of variant v at time t, multiplied by a proportionality constant.	Dynamically calculated during model run
χ	Force of infection proportionality constant	218 (baseline) (±5 for uncertainty)	Determined by calibration (Supporting Information, part 2)
σ	Duration of exposure	2 days	Kremer et al. 2022 (1)
γ	Duration of infection	2.2 days	Determined by calibration (Supporting Information, part 2), accounting for mean effects of isolation.
ω_1	Recovery duration from R_1 to R_2	90 days	Definition of compartment
ω_2	Recovery duration from R_2 to R_3	90 days	Definition of compartment
ω_3	Recovery duration from R_3 to R_4	180 days	Definition of compartment
ψ_1	Protection against reinfection 0-3 months since vaccination	74.8% (66.0%- 81.9%)	Bobrovitz et al. (2) 2023; Arabi et al. 2023 (3)
ψ_2	Protection against reinfection 3-6 months since vaccination	61.6% (51.2%- 71.1%)	Bobrovitz et al. (2) 2023; Arabi et al. 2023 (3)
ψ_3	Protection against reinfection 6-12 months since vaccination	13.0% (11.0%- 14.0%)	Bobrovitz et al. (2) 2023; Arabi et al. 2023 (3)
ψ_4	Protection against reinfection 12+ months since vaccination	13.0% (11.0%- 14.0%)	Bobrovitz et al. (2) 2023; Arabi et al. 2023 (3)
η^a	Probability of severe disease given COVID-19 infection for age category a ∈ {0-5 years, 6-17 years, 18-64 years, 65-79 years, 80+ years}	0.00288 for 0-5 years<br>0.00288 for 6-17 years<br>0.02052 for 18-64 years<br>0.07002 for 65-79 years<br>0.099 for 80+ years	Victorian Department of Health COVID-19 treatment data, available in previous Burnet Institute COVID-19 modelling reports (4, 5)

Symbol	Parameter	Value	Source
ξ_1	Protection against severe disease given infection 0-3 months since immune boosting event (vaccination or infection)	60%	Cromer et al. 2023 (6)
ξ_2	Protection against severe disease given infection 3-6 months since immune boosting event (vaccination or infection)	55%	Cromer et al. 2023 (6)
ξ_3	Protection against severe disease given infection 6-12 months since immune boosting event (vaccination or infection)	50%	Cromer et al. Nat Commun 2023 (6)
ξ_4	Protection against severe disease given infection 12+ months since immune boosting event (vaccination or infection)	40%	Cromer et al. 2023 (6)
ζ	Treatment efficacy against severe disease given infection	14%	Assuming treatment with molnupiravir (7)
θ^a	Treatment coverage in age category a ∈ {0-5 years, 6-17 years, 18-64 years, 65-79 years, 80+ years}	0% for 0-5 years<br>0% for 6-17 years<br>0% for 18-64 years<br>17.5% for 65-79 years<br>88.9% for 80+ years	Victorian Department of Health COVID-19 treatment data, available in previous Burnet Institute COVID-19 modelling reports (4, 5)
ν	Birth rate (per year)	97,500 (±2500 for uncertainty)	2022 Australian census, Victorian data (8)
μ^a	Non-COVID-19 mortality rate for age category a ∈ {0-5 years, 6-17 years, 18-64 years, 65-79 years, 80+ years}	0.00074 for 0-5 years<br>0.00015 for 6-17 years<br>0.00181 for 18-64 years<br>0.0175 for 65-79 years<br>0.1026 for 80+ years<br>(±5% for uncertainty)	Australian Institute of Health and Welfare (9)
μ_C^a	COVID-19 case fatality rate in the absence of prior immunity or treatment, for age category a ∈ {0-5 years, 6-17 years, 18-64 years, 65-79 years, 80+ years}	0.00002 for 0-5 years<br>0.00002 for 6-17 years<br>0.00032 for 18-64 years<br>0.0069 for 65-79 years<br>0.0554 for 80+ years<br>(±5% for uncertainty)	Knock et al. 2021 (10); Nyberg et al. 2022 (11)
α	Vaccination rate	Scenario dependent.	See section 1.6
ρ	Seeded cases used to initiate new variant when introduced.	10	Determined by calibration (Supporting Information, part 2)
ϕ^a	Rate of ageing out of age category a, for a ∈ {0-5 years, 6-17 years, 18-64 years, 65-79 years, 80+ years}	1/6 for 0-5 years<br>1/12 for 6-17 years<br>1/47 for 18-64 years<br>1/15 for 65-79 years<br>0 for 80+ years	Definition
N^a	Population size for age category a ∈ {0-5 years, 6-17 years, 18-64 years, 65-79 years, 80+ years}	382,727 for 0-5 years<br>1,190,902 for 6-17 years<br>3,953,396 for 18-64 years<br>812,897 for 65-79 years<br>286,042 for 80+ years	2022 Australian census, Victorian data (8)

1.2. Model dynamics and governing equations

Initial infection and recovery

People in the model are assumed to begin as susceptible (S), where they can become infected at a rate proportional to the dynamic prevalence of coronavirus disease 2019 (COVID-19) in the model (the force of infection is denoted λ, which is the dynamic prevalence multiplied by a proportionality constant χ). Following infection, people move to the exposed (E) compartment for an mean duration (σ) where they are not yet infectious to others, and then to the infected (I) compartment where they are infectious to others. After an mean duration of infection (γ), people move sequentially through a series of recovered compartments to approximate waning immunity over time (R_1 for an mean duration ω_1; R_2 for an mean duration ω_2; R_3 for an mean duration ω_3) before reaching a fourth recovered compartment (R_4) where they remain in the absence of further infection or vaccination, under the assumption that they retain some ongoing protection against reinfection or severe disease.

Reinfection

People can become reinfected from any of the recovered compartments and move back to the exposed compartment. However, the recovered compartments R_1, R_2, R_3, R_4 have infection risks reduced by factors ψ_1, ψ_2, ψ_3 and ψ_4 respectively, compared to those in the susceptible compartment. Waning of immunity over time is approximated by insisting that $\psi_1 \geq \psi_2 \geq \psi_3 \geq \psi_4$.

Vaccination

Vaccination is also included in the model at a rate (α) for people in the susceptible or recovered compartments. For simplicity, infection and vaccination are modelled to be equal "immune boosting events" that can wane over time, and hence when someone in the S compartment is vaccinated they move to the R_1 compartment (which has highest protection against infection), and when someone from the R_1, R_2, R_3 or R_4 compartments is vaccinated they also move to the R_1 compartment. Due to the high early vaccine coverage and the multiple COVID-19 waves in Victoria it is likely that most of the population has some form of hybrid immunity (i.e. vaccine-acquired plus exposure-acquired), and with limited data available on the distribution of immunity across the population it was not possible to stratify the model by immunity type. Therefore, for simplicity we have assumed the same probability of being reinfected following either vaccination or infection, and included a sensitivity analysis where vaccination is assumed to provide less protection than an infection (implemented as vaccinated people moving to the R_2 compartment rather than the R_1 compartment).

People in the susceptible compartment who are vaccinated are moved to the R1 compartment of the variant with highest current prevalence in the model. People who have been vaccinated or infected previously, and are currently in a recovered compartment associated with a particular variant, are moved into the R_1 compartment of that same variant when they are vaccinated (i.e. someone in a recovered compartment following infection with variant v_1 is moved into the R_1 compartment of variant v_1).

Stratification by age

The model compartments are stratified by an ordered set of age categories A = {0-5 years, 6-17 years, 18-64 years, 65-79 years, 80+ years}, denoted with the superscript $a \epsilon A$. Different age groups have different vaccination rates (α^a), vaccine eligibility, and risks of adverse outcomes following infection (see next subsection of this section). Based on 2024 Australian guidelines, people aged 18-64 years or 65+ years are only eligible for vaccination if it has been >12 months or >6 months respectively since their last immune boosting event (infection or vaccination), and people aged <18 years are not eligible for booster vaccine doses (12). For simplicity, homogeneous mixing is assumed between different age groups.

The model includes ageing between population groups, with ϕ^a representing the rate of ageing out of age group a (with ϕ^{80+} = 0 and ϕ^a defined as 0 for a<0).

Severity of outcomes following infection

The outcomes of infection were calculated outside of the model based on infections. The number of new severe cases was calculated by taking the number of new infections for each age category $a \in A$ and multiplying by the age-specific probability of a case being severe given infection (η^a). For infections among people in the recovered compartments, the probability of an infection being severe was reduced by the corresponding level of protection ($\xi_1, \xi_2, \xi_3, \xi_4$ for the R_1, R_2, R_3 and R_4 compartments respectively).

Whether or not a severe case resulted in hospitalisation depended on the coverage and effectiveness of COVID-19 treatment. For each age group $a \in A$, a proportion of severe cases were given treatment (θ^a) based off age-specific Victorian COVID-19 treatment data, which had an effectiveness (ζ) at preventing hospitalisation.

Stratification by COVID-19 variant

The E, I, and R compartments were also stratified by COVID-19 variant, denoted using superscript v, where $v \in V = \{v_1, v_2, \ldots, v_n\}$ corresponds to the n variants in the model (see section 1.3 for the number of variants in the implementation). For the E and I compartments this indicates current exposure or infection with a particular variant. For the recovered compartments, this is a record of which variant someone's most recent immunity was derived from (either through infection or through vaccination that was formulated around the same time). The model assumes each variant is an Omicron sub-variant with the same incubation period (σ), duration of infection (γ) and severity of outcomes.

A key feature that differentiates variants in the model is the relative cross-immunity between them (13). This is captured in the model using a cross-immunity parameter, where for $v_1, v_2 \in V$ the parameter $\kappa(v_1, v_2) \leq 1$ is defined as the risk of infection with variant v_2, for someone who has immunity from variant v_1, relative to their risk of infection with variant v_1. These cross-immunity parameters give newly introduced variants a competitive advantage in the model over existing circulating variants, and hence allows new variants to displace others and become dominant.

People in the susceptible and recovered compartments can be infected with any variant circulating in the model. For people in the susceptible compartment, the risk of infection with a variant $v \in V$ is proportional simply to the dynamic prevalence of the variant (the variant-specific force of infection is denoted $\lambda^v(t)$). For people in the recovered compartment associated with a specific variant $v_1 \in V$, the risk of infection with another variant $v_2 \in V$ in circulation is proportional to the dynamic prevalence of the variant (the variant-specific force of infection is denoted λ^{v_2}), the protection associated with the recovered compartment they are in (i.e. ψ_1 to ψ_4 for the R_1 to R_4 compartments respectively), and also to a cross immunity parameter $\kappa(v_1, v_2)$. For example, for someone in age group $a \in A$ who is 3-6 months post immune boosting event from variant v_1 (i.e. they are in the R_2^{a,v_1} compartment), the rate of infection with variant v_2 is proportional to $\lambda^{v_2} * \big(1 - \psi_2 * \kappa(v_1, v_2)\big)$.

Births and deaths

The model includes age-specific all-cause mortality (μ^a) and COVID-19 mortality (μ_C^a), and a birth rate (ν) that is fitted to maintain a constant model population (see table 1 and section 1.4).

Model equations

The model is governed by a set of ordinary differential equations. Let

$$\lambda^v(t) = \chi \frac{\sum_{a \in A} I^{a,v}}{\sum_{a \in A} N^a}$$

be the force of infection in the model for variant v, where:

$$N^a = S^a + \sum_{v \in V} (E^{a,v} + I^{a,v} + R_1{}^{a,v} + R_2{}^{a,v} + R_3{}^{a,v} + R_4{}^{a,v})$$

is the total model population in the age category a and χ is the force of infection proportionality constant.

The susceptible population is only stratified by age as it is not associated with any variant. The susceptible (S) compartment is defined by the following ordinary differential equation:

$$\frac{dS^a}{dt} = \nu - \sum_{w \in V} \lambda^w(t)\, S^a - \alpha^a S^a + \phi^{a-1} S^{a-1} - \phi^a S^a - \mu^a S^a$$

The remaining compartments are stratified by age and variant, and are governed by the following ordinary differential equations:

$$\begin{aligned}\frac{dE^{a,v}}{dt} = &\sum_{w \in V} \lambda^w(t)\, S^a + \sum_{w \in V} [(1 - \kappa(w,v)\psi_1)\lambda^v(t)]\, R_1{}^{a,w} + \sum_{w \in V} [(1 - \kappa(w,v)\psi_2)\lambda^v(t)]\, R_2{}^{a,w} \\ &+ \sum_{w \in V} [(1 - \kappa(w,v)\psi_3)\lambda^v(t)]\, R_3{}^{a,w} + \sum_{w \in V} [(1 - \kappa(w,v)\psi_4)\lambda^v(t)]\, R_4{}^{a,w} - \sigma E^{a,v} \\ &+ \phi^{a-1} E^{a-1,v} - \phi^a E^{a,v} - \mu^a E^{a,v}\end{aligned}$$

$$\frac{dI^{a,v}}{dt} = \sigma E^{a,v} - \gamma I^{a,v} + \phi^{a-1} I^{a-1,v} - \phi^a I^{a,v} - (\mu^a + \mu_C^a) I^{a,v}$$

$$\begin{aligned}\frac{dR_1{}^{a,v}}{dt} = &\gamma I^{a,v} - \omega_1 R_1^{a,v} - \sum_{w \in V} [(1 - \kappa(v,w)\psi_1)\lambda^w(t)]\, R_1{}^{a,v} + \alpha^a (S^a + R_3^{a,v} + R_4^{a,v}) \\ &+ \phi^{a-1} R_1^{a-1,v} - \phi^a R_1^{a,v} - \mu^a R_1^{a,v}\end{aligned}$$

$$\frac{dR_2{}^{a,v}}{dt} = \omega_1 R_1^{a,v} - \omega_2 R_2^{a,v} - \sum_{w \in V} [(1 - \kappa(v,w)\psi_2)\lambda^w(t)]\, R_2{}^{a,v} + \phi^{a-1} R_2^{a-1,v} - \phi^a R_2^{a,v} - \mu^a R_2^{a,v}$$

$$\begin{aligned}\frac{dR_3{}^{a,v}}{dt} = &\omega_2 R_2^{a,v} - \omega_3 R_3^{a,v} - \sum_{w \in V} [(1 - \kappa(v,w)\psi_3)\lambda^w(t)]\, R_3{}^{a,v} - \alpha^a R_3^{a,v} + \phi^{a-1} R_3^{a-1,v} - \phi^a R_3^{a,v} \\ &- \mu^a R_3^{a,v}\end{aligned}$$

$$\frac{dR_4{}^{a,v}}{dt} = \omega_3 R_3^{a,v} - \sum_{w \in V} [(1 - \kappa(v,w)\psi_4)\lambda^w(t)]\, R_4{}^{a,v} - \alpha^a R_4^{a,v} + \phi^{a-1} R_4^{a-1,v} - \phi^a R_4^{a,v} - \mu^a R_4^{a,v}$$

Note there is an asymmetry between the summations for people leaving the recovered compartments due to infection (they can be infected by any variant) and people entering the exposed compartment for variant v, where they could have been infected following exposure to any other variant.

Seeding cases

A new variant $v \in V$ is introduced by moving a number of people (ρ) from the infected compartment of the previously introduced variant into the infected compartment of the new variant ($I^{a,v}$). The non-zero prevalence of the variant v enables transmission to begin. The number of seeded cases used to introduce new variants (ρ) is determined through calibration (Supporting Information, part 2).

1.3. Model computational implementation

Having a large number of variants in the model is computationally challenging due to the number of compartments required, particularly for simulations run over many years. However, since new variants displace old ones relatively quickly, a simplification was used where the model is implemented using only four variants, with every fifth variant introduced re-using the first variant compartments. This is possible as after a variant has been out competed by four subsequent variants, there are few cases remaining in the model. The fractional number of people remaining in the Infected, Exposed and Recovered compartments are moved to the corresponding compartments of the variant that succeeded it, before seeding cases to initialize the new variant. For example, if the first, second, third and fourth variants were $v_1, v_2, v_3, v_4 \in V$, then to introduce the fifth variant, fractional remaining counts of people in the v_1 exposed, infected or recovered compartments would be moved to the corresponding compartments for v_2, and the new variant introduced using the v_1 compartments. This process then repeats for all further variants that are introduced.

1.4. Demographic characteristics

The model includes the entire population of Victoria, split into five age categories: 0-5, 6-17, 18-64, 65-79, 80+, based on 2022 census data for Victoria (Figure 1). The model population size assumed to be constant in scenarios since the results are intended to be applicable to the near future (achieved via fitting a birth rate, with the resulting total population in the model shown in
Figure 2.2).

The age categories in the model determine baseline vaccine coverage and eligibility for booster doses, and determine the probability that an infection is asymptomatic, mild, or severe. For simplicity we assume homogeneous mixing between age categories, which have been included to allow different age groups to be targeted in scenarios and for differential disease severity.

To generate uncertainty intervals for simulations, the initial population size is sampled from a distribution with the mean values in Figure 1 and a standard deviation of 10,000 persons per age category.

Figure 1. Victorian population size per age group in 2022 (8)

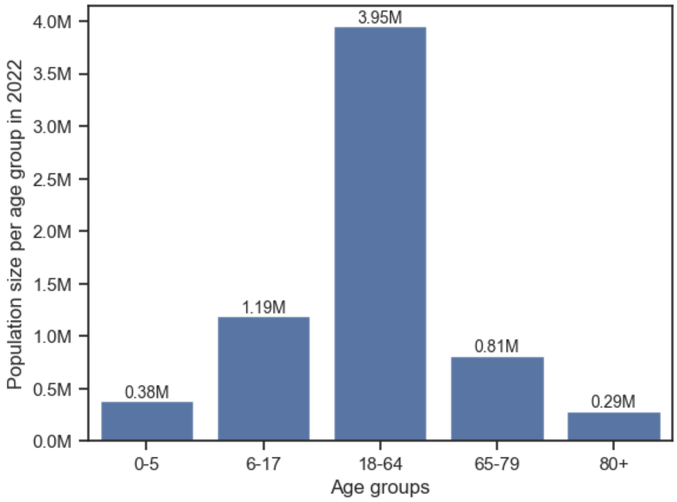

Figure 2. Model population over time given 6-monthly epidemic waves with uniform (spread across year) low vaccination coverage

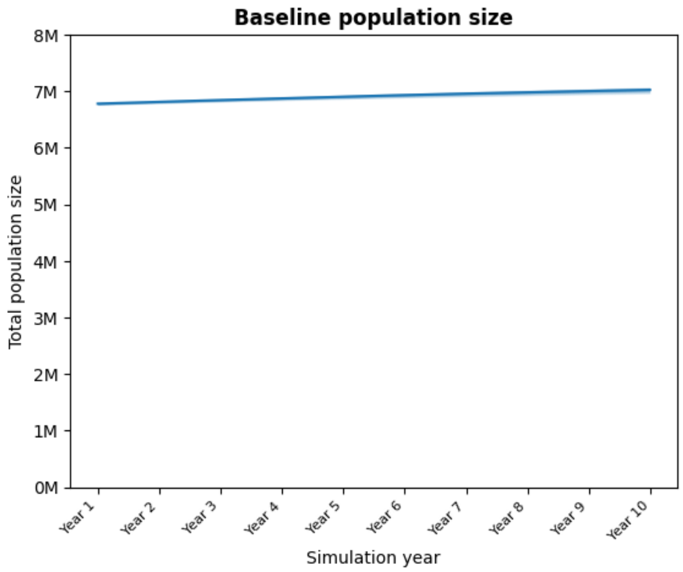

1.5. COVID-19 hospital admissions

In 2024 most Australian states ceased their hospital surveillance programs. Figure 3 shows the number of hospital admissions over time in Australia between January 2022 – January 2024 when the hospital surveillance program was still active. During 2023 there were 62,000 COVID-19 hospital admissions (14). The Victorian Department of Health reported 16,000 COVID-19-related hospitalisations in 2023 (4) (the Victoria population comprises about 25% of the Australian population).

Figure 3. COVID-19 hospital admissions for all of Australia between January 2022 - January 2024*

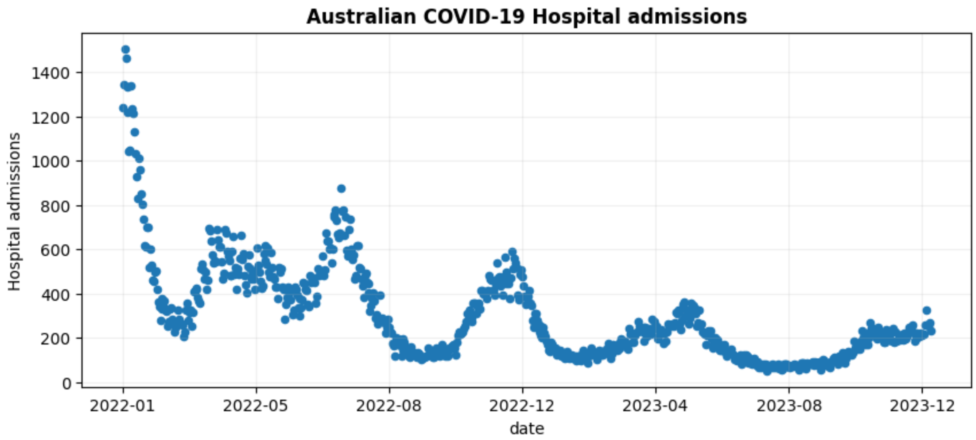

* Data source: Australian Department of Health and Aged Care (14).

1.6 Vaccination scenarios

For this analysis we use the annual vaccination coverage based on July 2024 data for the proportion of each age group who had received a vaccine in the past 12 months (15). The annual campaign vaccine scenarios assume a COVID-19 vaccine rollout period equal to the 2024 Victorian influenza vaccine rollout (Figure 4), while the non-campaign vaccination scenarios assume vaccines are delivered uniformly across the year.

Figure 4. Cumulative influenza vaccination coverage in Victoria, 2024*

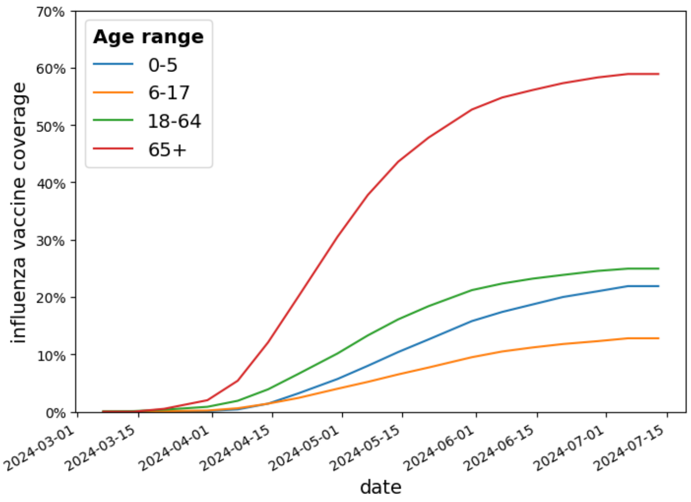

* Data source: Australian Government National Centre for Immunisation Research and Surveillance 2024 influenza vaccine coverage report (15).

2. Model calibration

The model baseline scenario includes epidemic waves of equal magnitude, with a peak every six months. These epidemic waves were generated by the entry of a new Omicron variant every six months which had reduced cross-immunity relative the previous variant (i.e. for people in the recovered compartments, immunity is less effective at preventing infection with the newly introduced variant).

The objective of the model calibration was to ensure that (1) the cumulative hospitalisations from each epidemic wave in the model aligned with the cumulative hospitalisations from the Omicron XBB wave that occurred in Victoria in 2022; (2) the peak daily hospital admissions in the model aligned with the peak daily hospital admissions from Omicron XBB; (3) the mean COVID-19 infection incidence per 100,000 person years aligned with estimates from previous Victorian model fitting exercises (4); and (4) the rate that new variants displaced old variants, calculated as the number of days the new variant took to go from 5% to 20% share of infections, was consistent with Victorian wastewater data showing the relative prevalence of a new variant over time.

To achieve this, an optimisation algorithm was used (a particle swarm optimisation algorithm from the Python *pyswam* library (16)), which varied the following parameters:

- Overall multiplier to the force of infection (χ).
- Variant relative cross immunity (Supporting Information, part 1); note that since we are modelling regular epidemic wave patterns, the calibration assumes that the cross-immunity advantage of each new variant is equal. For example, if $v_i, v_{i+1}, v_{i+2} \in V$ are a sequence of variants being introduced, then the cross-immunity $\kappa(v_i, v_{i+1})$ is equal to $\kappa(v_{i+1}, v_{i+2})$),
- Number of incursions for new variant (ρ).
- The probability of hospitalisation given severe COVID-19 in the absence of vaccination or prior infection.
- Duration of infection (γ). The rationale for varying this parameter is to include the population mean effects of isolation (i.e. the mean duration of infection represents the mean duration of time spent infecting others).

The optimisation aimed to minimise an objective function calculated as the fractional error of the modelled outputs compared to the real-world data. Within the objective function a higher weighting (3 times) was given to cumulative hospitalisations per epidemic wave and annual infection incidence per 100,000 person years compared to peak daily hospital admissions and the rate than new variants replaced old ones, as these measures were the closest related to the primary reported outcomes of this study. The final model runs using the calibrated parameters were compared with these historical takeover times to ensure the modelled takeover times lay within a realistic domain.

The resulting calibrated parameters are shown in Table 3 for each assumption about future epidemic wave patterns parameters. Only the 6-monthly future epidemic wave scenario was calibrated to hospital data, since the others are theoretical variations of this. However, each assumed epidemic wave pattern was compared against wastewater data to ensure the variant displacement time was without a reasonable range.

Table 3. Calibrated parameters for each future epidemic wave assumption

Future epidemic wave assumption	Force of infection multiplier	Variant relative cross immunity	Number of incursions	Probability of hospitalisation given severe COVID-19	Duration of infection (days)
**5-monthly waves**	218	0.1	30	0.21	2.2
**6-monthly waves**	218	0.2	30	0.21	2.2
**8-monthly waves**	218	0.2	30	0.21	2.2
**Annual waves**	218	0.2	30	0.21	2.2
**Shorter than XBB**	218	0.35	30	0.21	2.2
**Taller than XBB**	230	0.0	30	0.21	2.2

Supplementary results

3. Simulation length

Due to the variation in future epidemic wave periodicity and magnitude, the model proved highly sensitive to start or end of the measurement period. The model was run for 1-, 5- and 10-years with different simulation lengths tested to investigate the impact on the simulation length. For 1- and 5-years (figure 10, figure 11) the results proved sensitive to the start time chose, but for a 10-year projection period the results converged and were less sensitive to the start or end points chosen (figure 12).

Figure 5. Proportional difference in annual hospitalisation incidence between the baseline and annual vaccination starting in August, given different simulation lengths between 1- and 2-years

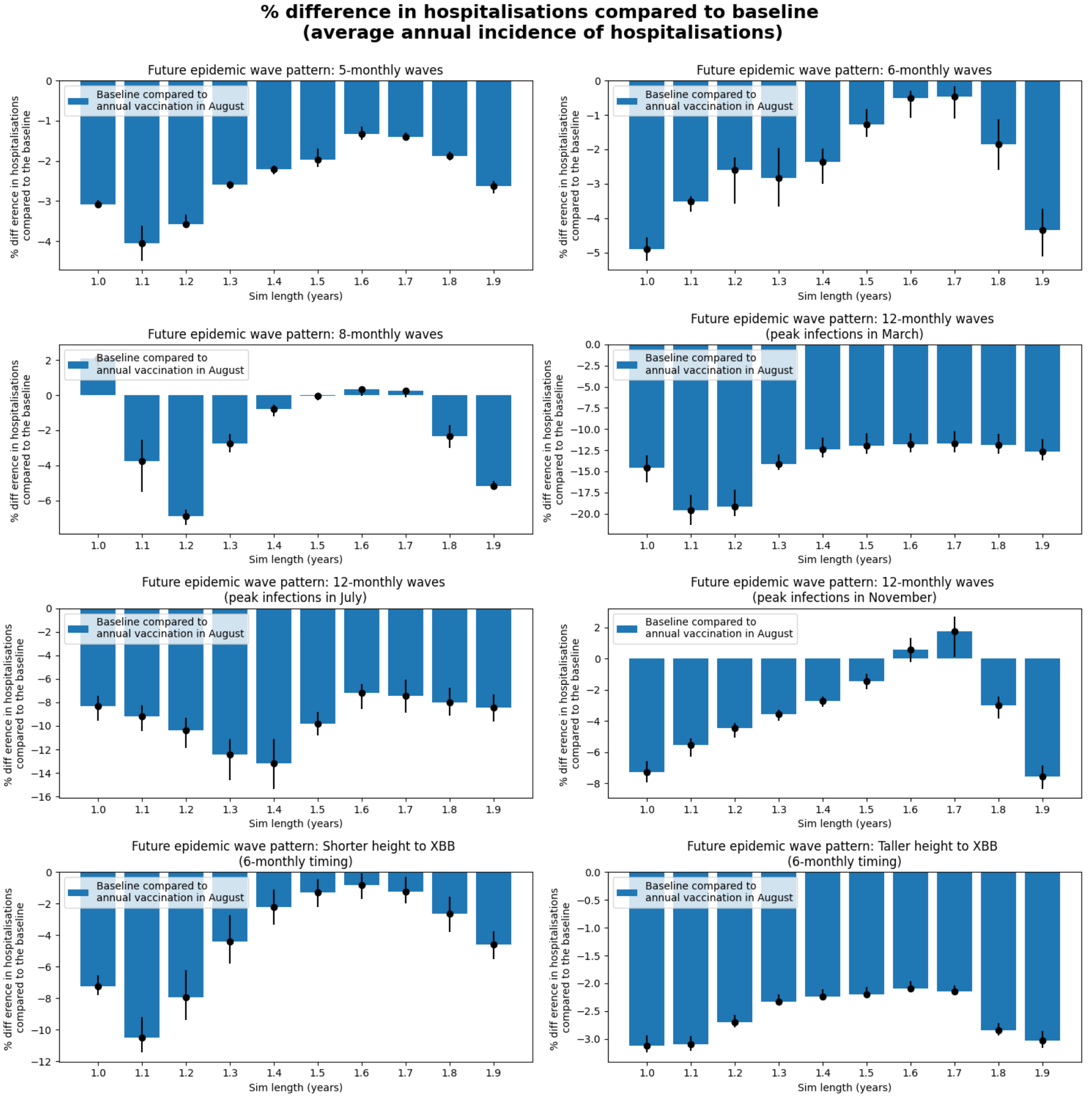

Figure 6. Proportional difference in annual hospitalisation incidence between the baseline and annual vaccination starting in August, given different simulation lengths between 5- and 6-years

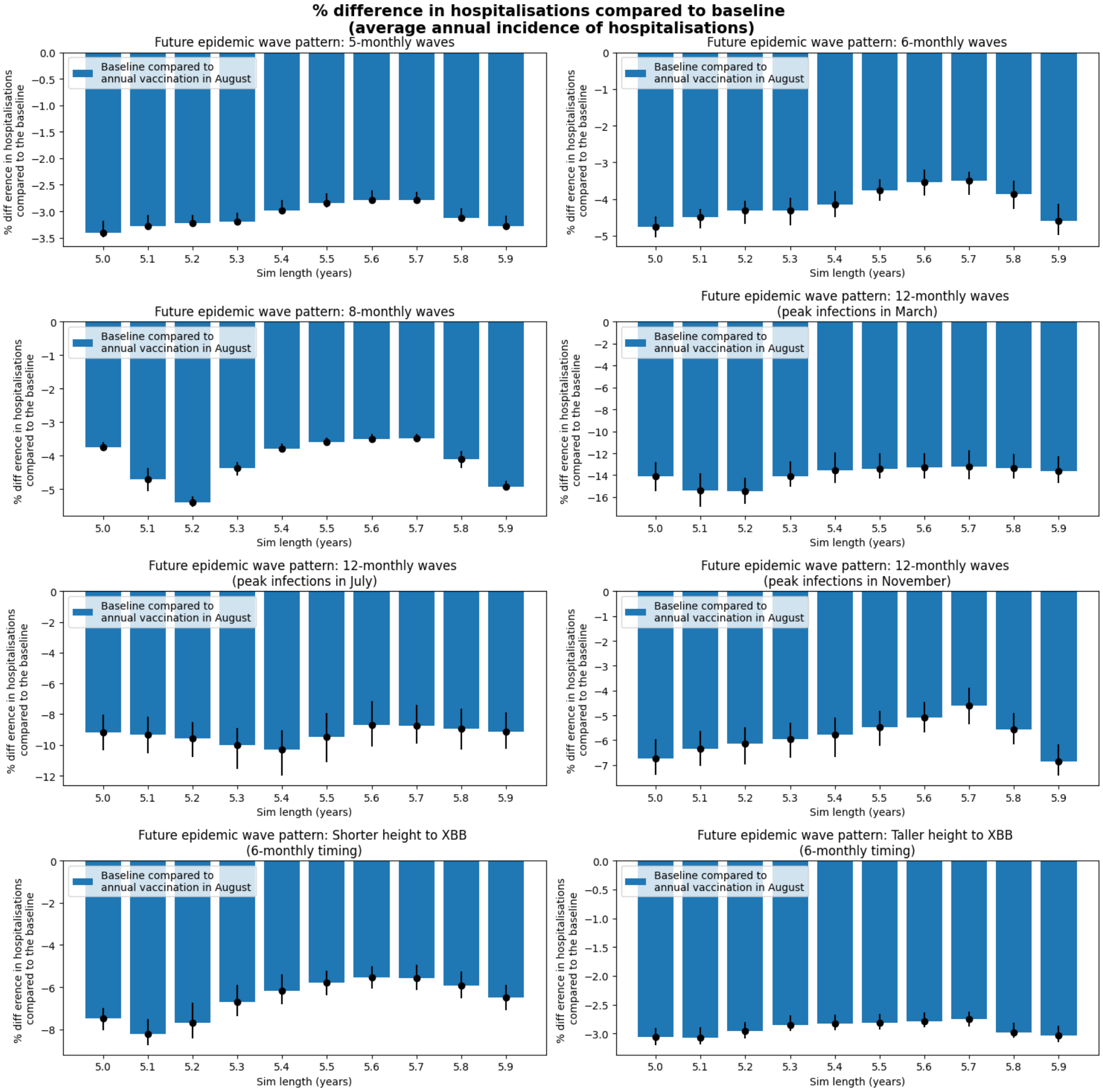

Figure 7. Proportional difference in annual hospitalisation incidence between the baseline and annual vaccination starting in August, given different simulation lengths between 10- and 11-years

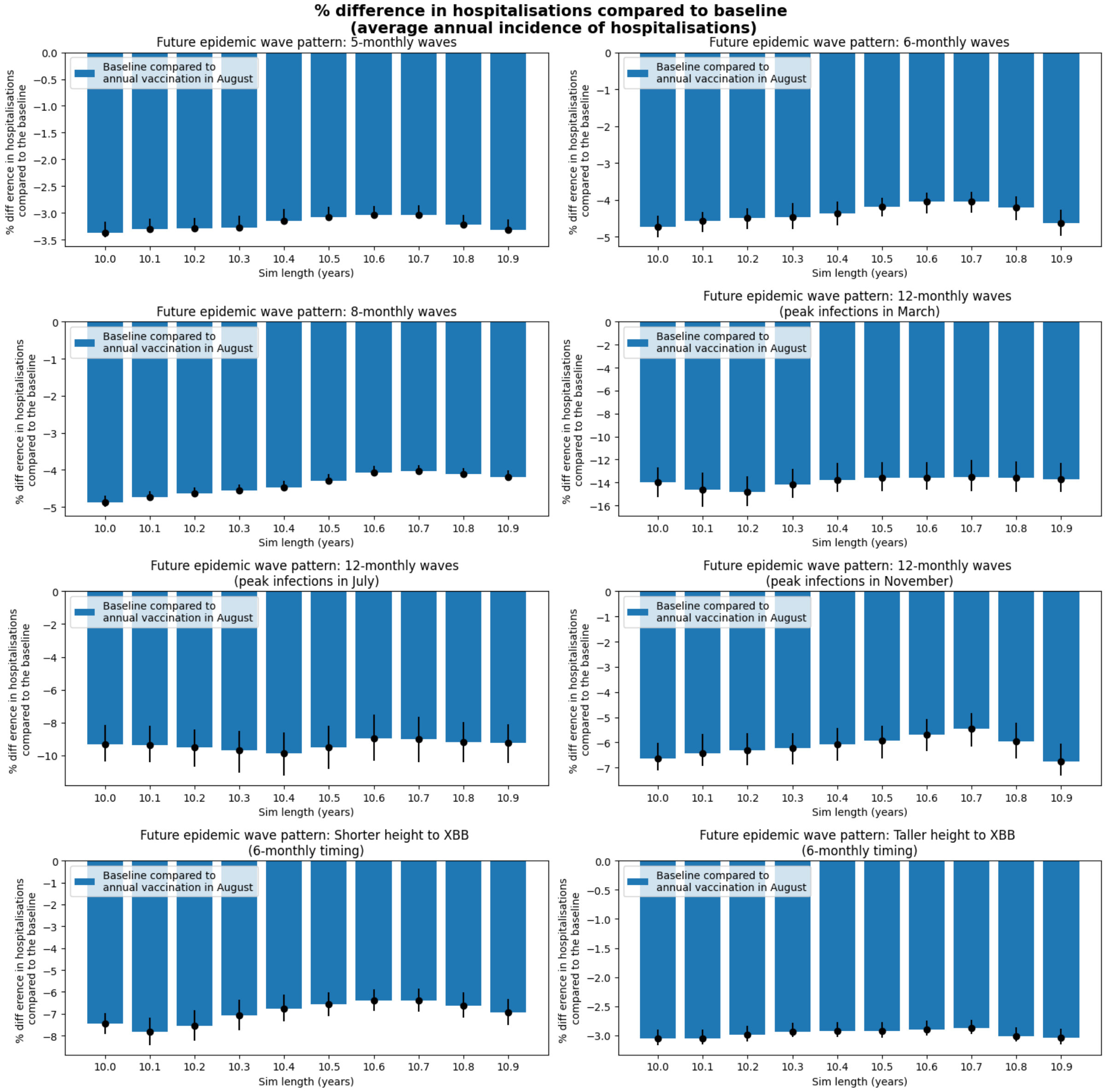

3.3. Epidemic wave patterns

All vaccination scenarios were run with several sets of different assumptions regarding the periodicity and magnitude of epidemic waves. Figures 11 and 12 display new infections new hospitalisations respectively over a 10-year period for each modelled future epidemic wave scenario

Figure 8. Daily new infections over a 10-year period for different future epidemic wave patterns

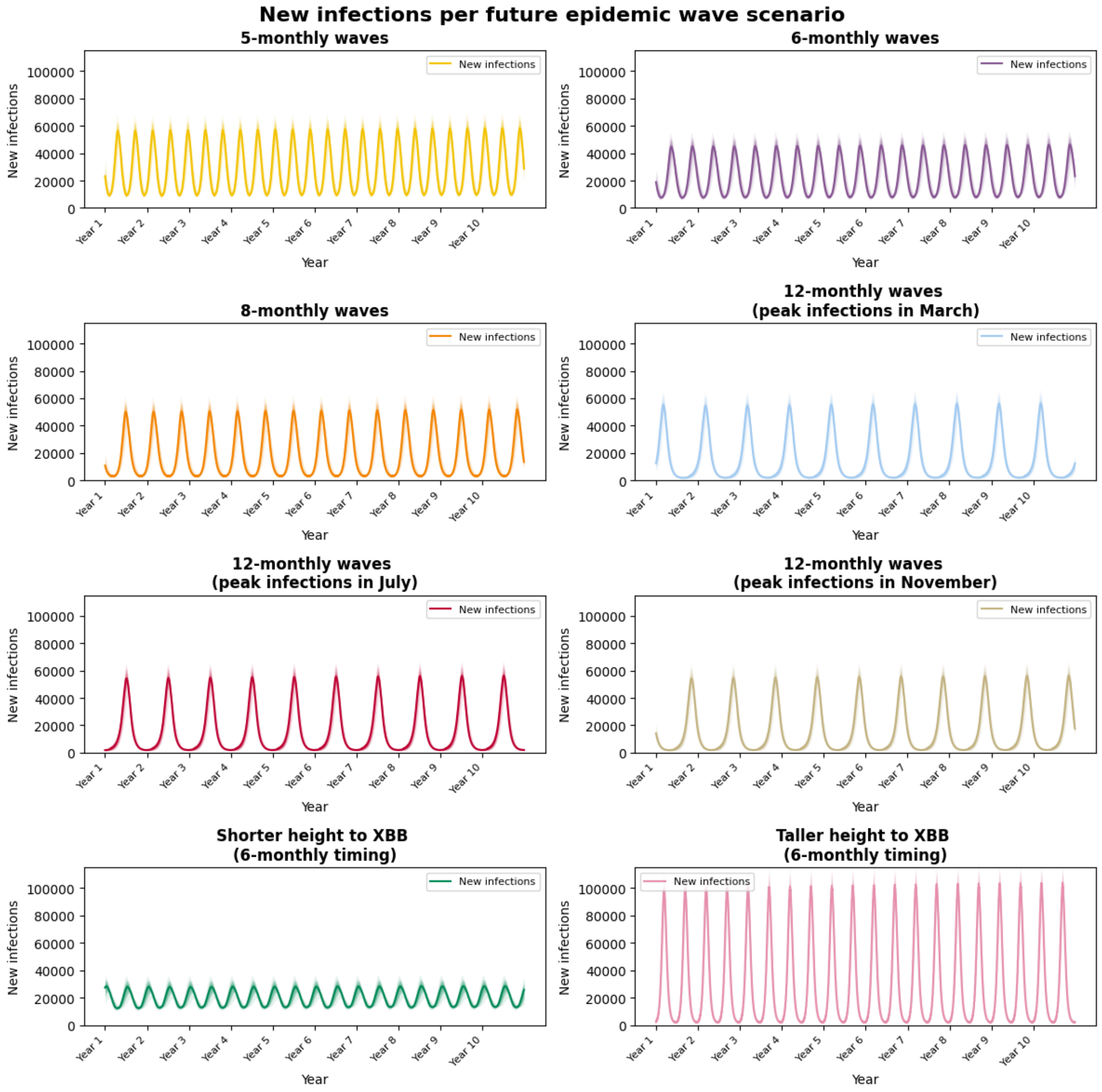

Figure 9. Daily new hospitalisations over a 10-year period for different future epidemic wave patterns

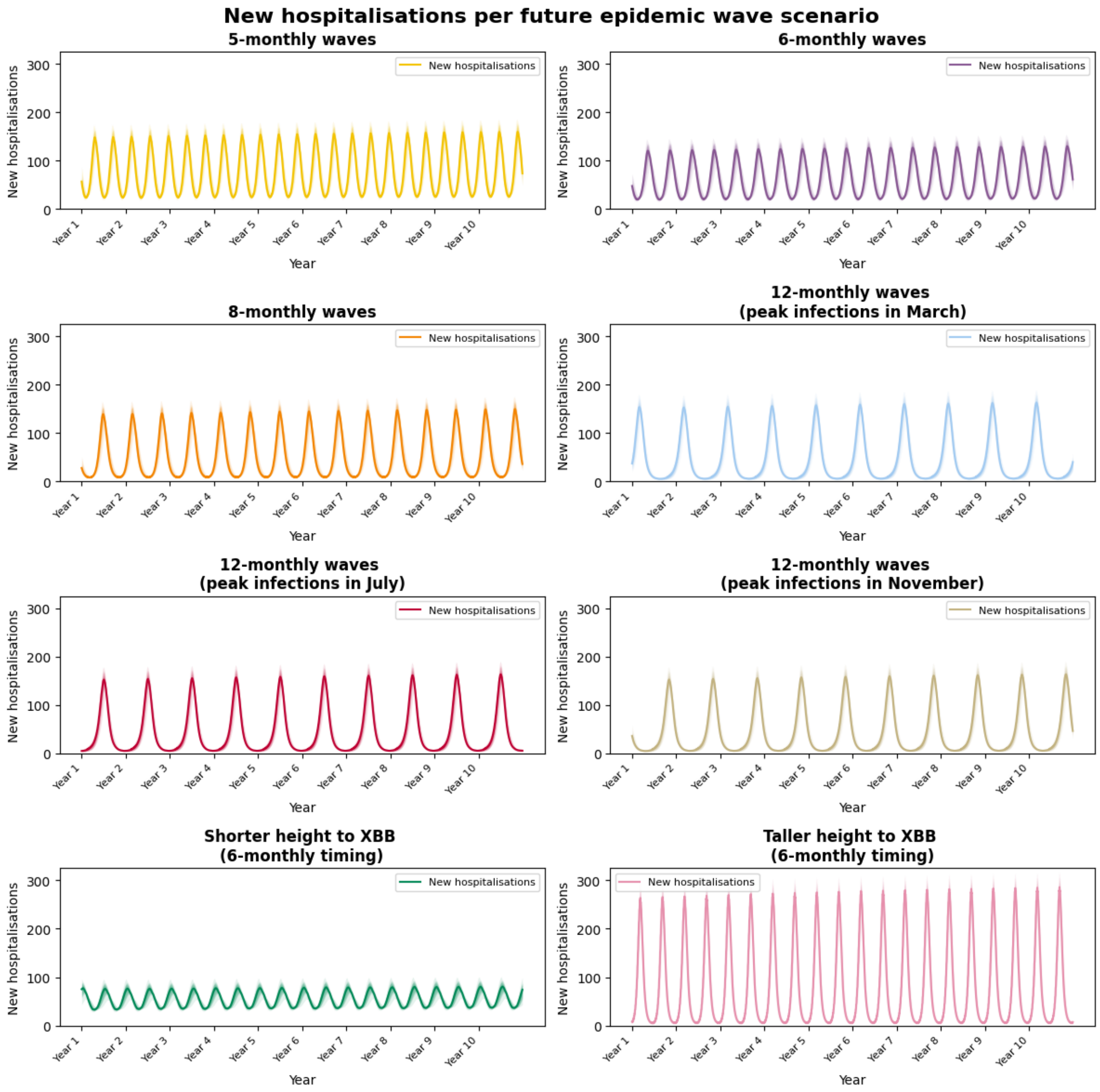

Table 4. Proportional changes (with 95% confidence interval) in projected median annual incidence of SARS-CoV-2 infections per 100 000 person-years, compared with baseline, by vaccination scenario and epidemic wave characteristics

	Interval between COVID-19 waves					
**Scenario**	**6 months**	**5 months**	**8 months**	**12 months (March peak)**	**12 months (July peak)**	**12 months (November peak)**
Baseline (number of infections per 100 000 person-years)	118 288 (104 221–143 107)	150 243 (134 188–178 415)	93 879 (83 476–114 029)	78 002 (63 547–97 928)	78 184 (63 695–97 983)	78 176 (63 592–98 023)
High coverage, vaccinations spread across year	–15% (–15% to –14%)	–7% (–7% to –6%)	–17% (–17% to –15%)	–31% (–32% to –29%)	–31% (–32% to –29%)	–31% (–32% to –29%)
Increased coverage for people aged 65 years or more	–6% (–7% to –5%)	–3% (–4% to –3%)	–7% (–8% to –6%)	–18% (–20% to –17%)	–18% (–20% to –16%)	–18% (–20% to –16%)
Coverage for people under 65 years of age doubled	0% (0% to 0%)	0% (0% to 0%)	0% (0% to 0%)	–2% (–2% to –2%)	–2% (–2% to –2%)	–2% (–2% to –2%)
Annual vaccination campaign starting in March*	–3% (–3% to –3%)	–1% (–1% to –1%)	–2% (–2% to –2%)	–10% (–12% to –9%)	–1% (–2% to 0%)	–13% (–14% to –12%)
Annual vaccination campaign starting in August	–2% (–2% to –2%)	–1% (–1% to –1%)	–2% (–2% to –2%)	–13% (–15% to –11%)	–10% (–11% to –8%)	–1% (–2% to 0%)
Annual vaccination campaign starting in December	–2% (–3% to –2%)	–1% (–1% to –1%)	–2% (–2% to –2%)	–1% (–2% to 0%)	–13% (–14% to –12%)	–10% (–11% to –8%)
Coverage equivalent to influenza vaccination coverage, vaccinations spread across year	–2% (–2% to –1%)	–1% (–1% to –1%)	–2% (–2% to –2%)	–5% (–6% to –4%)	–5% (–6% to –4%)	–5% (–6% to –4%)
No vaccination for people under 65 years of age	1% (1% to 1%)	0% (0% to 0%)	1% (1% to 1%)	2% (2% to 2%)	2% (2% to 2%)	2% (2% to 2%)
No COVID-19 vaccinations	2% (2% to 2%)	1% (1% to 1%)	3% (2% to 3%)	8% (8% to 9%)	8% (8% to 9%)	8% (8% to 9%)

CI = confidence interval; COVID-19 = coronavirus disease 2019; SARS-CoV-2 = severe acute respiratory syndrome coronavirus 2.

* That is, same time as influenza vaccination campaign.

Table 5. Proportional changes (with 95% confidence interval) in projected median annual incidence of COVID-19-related hospitalisations per 100 000 person-years, compared with baseline, by vaccination scenario and epidemic wave characteristics

	Interval between COVID-19 waves					
**Scenario**	**6 months**	**5 months**	**8 months**	**12 months (March peak)**	**12 months (July peak)**	**12 months (November peak)**
Baseline (number of COVID-19-related hospitalisations per 100 000 person-years)	326 (288–383)	403 (361–467)	264 (236–313)	222 (183–270)	223 (185–272)	223 (184–271)
High coverage, vaccinations spread across year	–26% (–26% to –24%)	–16% (–17%, –15%)	–29% (–30%, –28%)	–42% (–43%, –40%)	–42% (–43%, –39%)	–42% (–43%, –40%)
Increased coverage for people aged 65 years or more	–14% (–14% to –13%)	–9% (–10%, –9%)	–15% (–16%, –15%)	–26% (–27%, –24%)	–26% (–27%, –24%)	–26% (–27%, –24%)
Coverage for people under 65 years of age doubled	0% (0% to 0%)	0% (0%, 0%)	0% (0%, 0%)	–2% (–2%, –1%)	–2% (–2%, –1%)	–2% (–2%, –1%)
Annual vaccination campaign starting in March*	–6% (–6% to –5%)	–3% (–3%, –3%)	–5% (–5%, –5%)	–10% (–11%, –9%)	–6% (–7%, –6%)	–14% (–15%, –13%)
Annual vaccination campaign starting in August	–5% (–5% to –4%)	–3% (–3%, –3%)	–5% (–5%, –5%)	–14% (–15%, –13%)	–9% (–11%, –8%)	–7% (–7%, –6%)
Annual vaccination campaign starting in December	–4% (–5% to –4%)	–3% (–3%, –3%)	–5% (–5%, –5%)	–7% (–7%, –6%)	–14% (–15%, –13%)	–9% (–10%, –8%)
Coverage equivalent to influenza vaccination coverage, vaccinations spread across year	–2% (–2% to –2%)	–1% (–1%, –1%)	–2% (–2%, –2%)	–5% (–5%, –4%)	–5% (–5%, –4%)	–5% (–5%, –4%)
No vaccination for people under 65 years of age	1% (1% to 1%)	1% (1%, 1%)	1% (1%, 1%)	2% (2%, 2%)	2% (2%, 2%)	2% (2%, 2%)
No COVID-19 vaccinations	6% (6% to 7%)	5% (4%, 5%)	7% (7%, 7%)	13% (12%, 13%)	13% (12%, 13%)	13% (12%, 13%)

CI = confidence interval; COVID-19 = coronavirus disease 2019.

* That is, same time as influenza vaccination campaign.

4. Sensitivity analyses

4.1. Vaccine efficacy

As a sensitivity analysis we compared our assumption in the main analysis that vaccination and infection offer the same protection, to an alternate assumption where vaccination provides less protection than an infection (implemented as vaccinated people moving to the R_2 compartment rather than the R_1 compartment). For this test we considered the impact of annual vaccination starting in August compared to the baseline scenario of low uniform coverage given a 6-monthly epidemic wave pattern (Figure 7).

If vaccination offered less protection than infection, this resulted in a smaller impact of the scenarios, with a 2% and 4% reduction in the mean incidence of infections and hospitalisations respectively, compared to the main analysis which had a reduction in mean incidence of infections and hospitalisations of 3.5% and 6% respectively.

Figure 10. Proportional difference in annual incidence of infections and hospitalisations per 100,000 person years over a 10-year projection period compared to the baseline for annual vaccination starting in August with future epidemic waves occurring every 6-months. Bars are coloured by vaccine efficacy

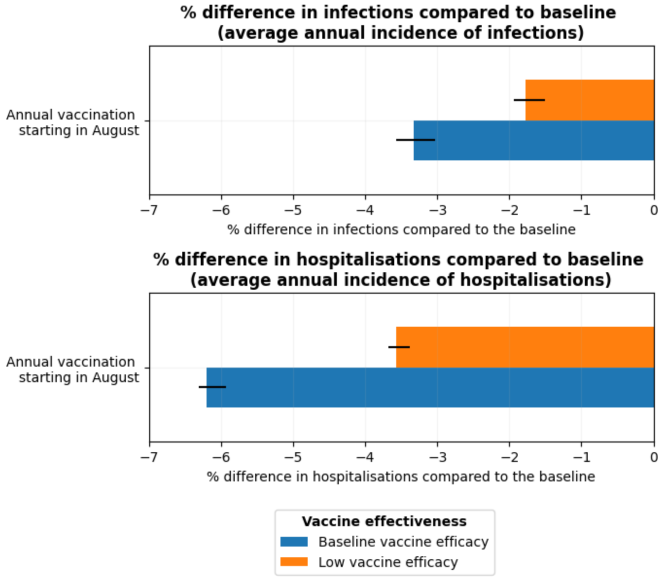

4.2. Epidemic wave magnitude

Figure 11. Proportional changes (with 95% confidence interval) in projected median annual incidence of SARS-CoV-2 infections per 100 000 person-years, compared with baseline, by vaccination scenario and epidemic wave infection peak height

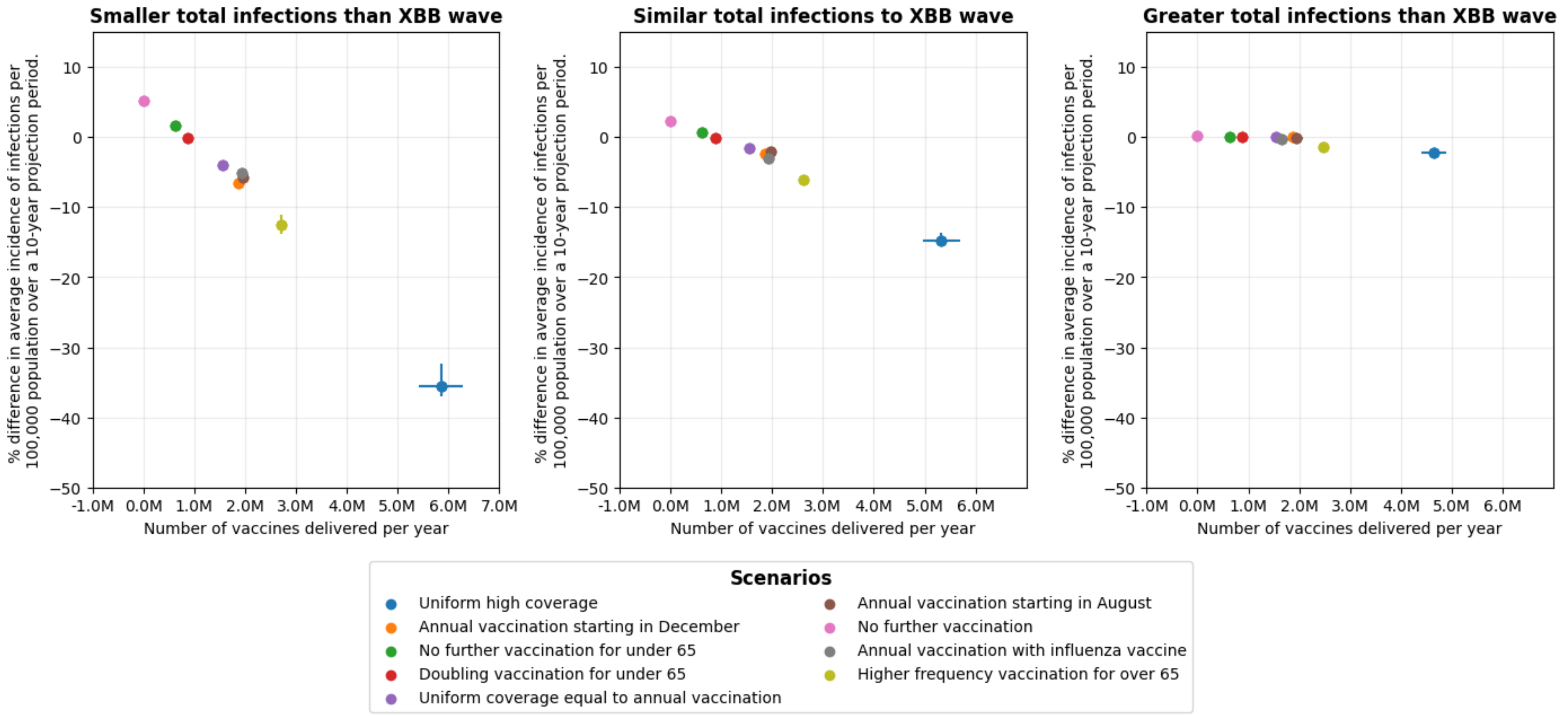

* The proportional differences data for these graphs are included in table 6.

Table 6. Proportional changes (with 95% confidence interval) in projected median annual incidence of SARS-CoV-2 infections per 100 000 person-years, compared with baseline, by vaccination scenario and epidemic wave infection peak height

	Interval between COVID-19 waves, height of infections peak		
**Scenario**	**6 months, peak infections same as XBB wave**	**6 months, peak infections 30% lower**	**6 months, peak infections 15% higher**
Baseline (number of infections per 100 000 person-years)	118,288 (104,221–143,107)	101,910 (85,048–126,856)	155,099 (143,674–178,203)
High coverage, vaccinations spread across year	–15% (–15% to –14%)	–36% (–37% to –32%)	–2% (–2% to –2%)
Increased coverage for people aged 65 years or more	–6% (–7% to –5%)	–13% (–14% to –11%)	–1% (–2% to –1%)
Coverage for people under 65 years of age doubled	0% (0% to 0%)	0% (0% to 0%)	0% (0% to 0%)
Annual vaccination campaign starting in March*	–3% (–3% to –3%)	–5% (–5% to –5%)	0% (0% to 0%)
Annual vaccination campaign starting in August	–2% (–2% to –2%)	–6% (–6% to –5%)	0% (0% to 0%)
Annual vaccination campaign starting in December	–2% (–3% to –2%)	–7% (–7% to –6%)	0% (0% to 0%)
Coverage equivalent to influenza vaccination coverage, vaccinations spread across year	–2% (–2% to –1%)	–4% (–5% to –4%)	0% (0% to 0%)
No vaccination for people under 65 years of age	1% (1% to 1%)	2% (1% to 2%)	0% (0% to 0%)
No COVID-19 vaccinations	2% (2% to 2%)	5% (5% to 5%)	0% (0% to 0%)

CI = confidence interval; COVID-19 = coronavirus disease 2019.

* That is, same time as influenza vaccination campaign.

Figure 12. Proportional changes (with 95% confidence interval) in projected median annual incidence of COVID-19-related hospitalisations per 100 000 person-years, compared with baseline, by vaccination scenario and epidemic wave infection peak height

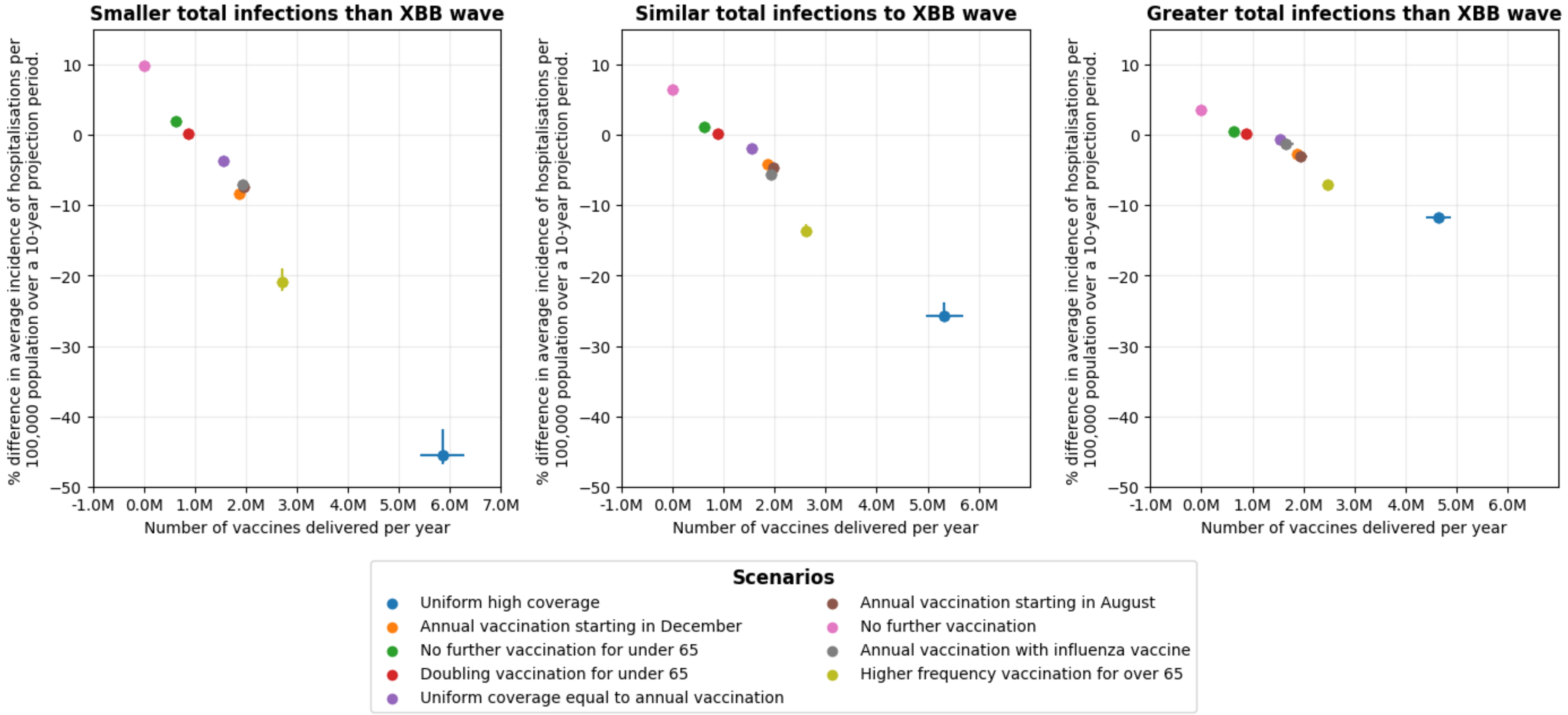

* The proportional differences data for these graphs are included in table 7.

Table 7. Proportional changes (with 95% confidence interval) in projected median annual incidence of COVID-19-related hospitalisations per 100 000 person-years, compared with baseline, by vaccination scenario and epidemic wave infection peak height

	Interval between COVID-19 waves, height of infections peak		
**Scenario**	**6 months, peak infections same as XBB wave**	**6 months, peak infections 30% lower**	**6 months, peak infections 15% higher**
Baseline (number of COVID-19-related hospitalisations per 100 000 person-years)	326 (288–383)	283 (238–342)	415 (386–468)
High coverage, vaccinations spread across year	–15% (–15% to –14%)	–45% (–47% to –42%)	–12% (–12% to –11%)
Increased coverage for people aged 65 years or more	–6% (–7% to –5%)	–21% (–22% to –19%)	–7% (–7% to –7%)
Coverage for people under 65 years of age doubled	0% (0% to 0%)	0% (0% to 0%)	0% (0% to 0%)
Annual vaccination campaign starting in March*	–3% (–3% to –3%)	–7% (–7% to –7%)	–1% (–1% to –1%)
Annual vaccination campaign starting in August	–2% (–2% to –2%)	–7% (–8% to –7%)	–3% (–3% to –3%)
Annual vaccination campaign starting in December	–2% (–3% to –2%)	–8% (–9% to –8%)	–3% (–3% to –3%)
Coverage equivalent to influenza vaccination coverage, vaccinations spread across year	–2% (–2% to –1%)	–4% (–4% to –3%)	–1% (–1% to –1%)
No vaccination for people under 65 years of age	1% (1% to 1%)	2% (2% to 2%)	0% (0% to 0%)
No COVID-19 vaccinations	2% (2% to 2%)	10% (9% to 10%)	4% (3% to 4%)

CI = confidence interval; COVID-19 = coronavirus disease 2019.

* That is, same time as influenza vaccination campaign.

References

1. Kremer C, Braeye T, Proesmans K, et al. Serial intervals for SARS-CoV-2 Omicron and Delta Variants, Belgium, November 19-December 31, 2021. Emerg Infect Dis 2022; 28: 1699-1702.
2. Bobrovitz N, Ware H, Ma X, et al. Protective effectiveness of previous SARS-CoV-2 infection and hybrid immunity against the omicron variant and severe disease: a systematic review and meta-regression. Lancet Infect Dis 2023; 23: 556-567.
3. Arabi M, Al-Najjar Y, Sharma O, et al. Role of previous infection with SARS-CoV-2 in protecting against omicron reinfections and severe complications of COVID-19 compared to pre-omicron variants: a systematic review. BMC Infect Dis 2023; 23: 432.
4. McAndrew F, Abeysuriya RG, Sacks-Davis R, et al. Admission screening testing of patients and staff N95 respirators are cost-effective in reducing COVID-19 hospital-acquired infections. J Hosp Infect 2024; 152: 81-92.
5. McAndrew F, Sacks-Davis R, Abeysuriya R, et al. COVID-19 outbreaks in Residential Aged Care Facilities: an agent-based modelling study. Front Public Health 2024; 12: e1344916.
6. Cromer D, Steain M, Reynaldi A, et al. Predicting vaccine effectiveness against severe COVID-19 over time and against variants: a meta-analysis. Nat Commun 2023; 14: 1633.
7. Wong CKH, Au ICH, Lau KTK, et al. Real-world effectiveness of molnupiravir and nirmatrelvir plus ritonavir against mortality, hospitalisation, and in-hospital outcomes among community-dwelling, ambulatory patients with confirmed SARS-CoV-2 infection during the omicron wave in Hong Kong: an observational study. Lancet 2022; 400: 1213-1222.
8. Australian Bureau of Statistics. Regional population by age and sex. 29 Aug 2024. https://www.abs.gov.au/statistics/people/population/regional-population-age-and-sex/latest-release (viewed Apr 2025).
9. Australian Institute of Health and Welfare. Deaths in Australia. Updated 9 Apr 2025. https://www.aihw.gov.au/reports/life-expectancy-deaths/deaths-in-australia/contents/age-at-death (viewed Apr 2025).
10. Knock ES, Whittles LK, Lees JA, et al. Key epidemiological drivers and impact of interventions in the 2020 SARS-CoV-2 epidemic in England. Sci Transl Med 2021; 13: e4262.
11. Nyberg T, Ferguson NM, Nash SG, et al. Comparative analysis of the risks of hospitalisation and death associated with SARS-CoV-2 omicron (B.1.1.529) and delta (B.1.617.2) variants in England: a cohort study. Lancet 2022; 399: 1303-1312.
12. Australian Department of Health and Aged Care. COVID-19 vaccine advice and recommendations for 2024. Updated 20 Nov 2024. https://www.health.gov.au/our-work/covid-19-vaccines/getting-your-vaccination/booster-doses (viewed Apr 2025).
13. Thompson RN, Southall E, Daon Y, et al. The impact of cross-reactive immunity on the emergence of SARS-CoV-2 variants. Front Immunol 2022; 13: e1049458.
14. Australian Department of Health and Aged Care. COVID-19 reporting. Updated 17 Oct 2024. Archived: https://web.archive.org/web/20241105022219/https://www.health.gov.au/topics/covid-19/reporting (viewed Apr 2025).
15. National Centre for Immunisation Research and Surveillance. Historical influenza vaccine coverage (%) at end of year, by age group, Australia, 2020–2024. https://ncirs.org.au/influenza-vaccination-coverage-data/all-persons-2020-2025-ytd-influenza-vaccination-coverage (viewed Apr 2025).
16. Miranda LJ. PySwarms: a research toolkit for Particle Swarm Optimization in Python. Journal of Open Source Software 2018; 3: 433.
